# Resource competition and technological diversity

**DOI:** 10.1371/journal.pone.0259875

**Published:** 2021-11-18

**Authors:** Almaz Mustafin, Aliya Kantarbayeva

**Affiliations:** 1 Satbayev University, Almaty, Kazakhstan; 2 al-Farabi Kazakh National University, Almaty, Kazakhstan; Meiji University School of Interdisciplinary Mathematical Science, JAPAN

## Abstract

The work develops and investigates a mathematical model for evolution of the technological structure of an economic system where different technologies compete for the common essential resources. The model is represented by a system of consumer–resource rate equations. Consumers are technologies formalized as populations of weakly differentiated firms producing a similar commodity with like average output. Firms are characterized by the Leontief–Liebig production function in stock-flow representation. Firms self-replicate with a rate proportional to production output of the respective technology and dissolve with a constant rate of decay. The resources are supplied to the system from outside and consumed by concerned technologies; the unutilized resource amounts are removed elsewhere. The inverse of a per firm break-even resource availability is proposed to serve as a measure for competitiveness towards a given resource. The necessary conditions for coexistence of different technologies are derived, according to which each contender must be a superior competitor for one specific resource and an inferior competitor for the others. The model yields a version of the principle of competitive exclusion: in a steady state, the number of competing technologies cannot exceed the number of limiting resources. Competitive outcomes (either dominance or coexistence) in the general system of multiple technologies feeding on multiple essential resources are shown to be predictable from knowledge of the resource-dependent consumption and growth rates of each technological population taken alone. The proposed model of exploitative competition with explicit resource dynamics enables more profound insight into the patterns of technological change as opposed to conventional mainstream models of innovation diffusion.

## Introduction

The pioneering empirical works on technology adoption initiated by Ryan and Gross [1] and taken up by Griliches [2] induced a steady stream of studies aiming at describing the *diffusion*, i. e. spread, of technological innovations that has persisted to this day and remained highly topical. In economics terms, according to Stoneman [3], “Technological diffusion is the process by which innovations (be they new products, new processes or new management methods) spread within and across economies.” Innovation in this definition is understood in the sense of Schumpeter [4, p. 66] whose classical notion of the term encompasses (i) a new product or a new quality of the existing product; (ii) a new production method; (iii) a new market; (iv) a new source of supply; and (v) a reorganization in the industry, e. g. by merging or splitting. It is worth remarking that in his original writings Schumpeter refers to innovation as “neue Kombination” (new combination) [5], whose significance will become clear in what follows.

The extent of use of a new technology can be measured in a number of ways, three of which are most common: (i) absolute total usage/ownership at a given instant of time; (ii) usage/ownership relative to some total output measure, e. g. GDP; and (iii) total usage relative to some estimated post-diffusion (asymptotic or saturation) level of utilization [6]. Early and subsequent studies [7–12] found that the key temporal features of diffusion followed the typical pattern of a constrained *S*-shaped curve. Diffusion rates first increase and then decrease over time, featuring a phase of relatively rapid adoption sandwiched between an early interval of slow growth and a late segment of decelerated approach to saturation. Different theoretical approaches have been pursued to describe and give the rationale behind the main characteristics of the diffusion process. A large body of research is represented by regression models. Those are rather confirmatory than explanatory. To carry out regression analysis, a suitable form of the diffusion function must be specified. Once the relationship between the variables is decided, the problem is reduced to the estimation of the parameters by fitting the available information about these variables. Regression analysis is widely used for predicting and forecasting. Most of the available regression models are basically extensions, generalizations or combinations of the growth laws originally proposed by Verhulst [13], Bass [14], and Gompertz [15]. Inasmuch as the present research is not directly concerned with the validation of diffusion models by the instruments of regression analysis, we refer the reader to comprehensive surveys [12, 16–20] for more information in that direction.

The dominant explanatory models of diffusion are *epidemic models* based on an analogy between the spread of contagious diseases and that of technological novelty. According to a widely accepted definition given by Rogers [10, p. 35], “Diffusion is the process by which an innovation is communicated through certain channels over time among the members of a social system”. This interpretation, reflecting the epidemic standpoint, considers spread of a new technology to be fundamentally a social process with no direct relation to any business operation. The leading alternative to epidemic models is a *probit model* suggested by David [21] (ref. also [22]) which argues that firms, potential users of a new technology, justify their decisions on cost/benefit analysis. Probit models however are not sufficiently simple in structure to be analytically tractable. In the present paper, to meet the immediate objectives of our analysis, we focus on *population-based* approach (of which epidemic models are a part), as that well suits the major direction of our research. As Geroski [17] put it, “…there is not much choice between population and probit models. There are no drivers of diffusion which feature in population models which cannot be expressed one way or the other in probit form. And …population models can be extended to allow for heterogeneous populations defined by differences in some characteristic. The really interesting choice, I think, is between different types of population models …”.

Basically, epidemic approach distinguishes the two types of potential adopters of a new technology: those who are influenced in their decisions by mass-media on the one hand and word-of-mouth communication on the other. These categories are characterized as *innovative* and *imitative*, respectively. In a model by Fourt and Woodlock [23] only external influence on potential adopters is taken into account, and the interactions among the members of the social system are ignored. Let *N*(*t*) be a measure of use of the technology at time *t*. The rate of adoption, $\dot{N}$ (hereafter overdots will mean differentiation with respect to time), is assumed proportional to the frequency of contacts of the current number of potential adopters, (*K* − *N*), with an external source of information such as print media, radio, TV, Internet, outdoor advertising, independent mediators (instructors, consultants, commercial travellers, salespersons, etc.). Here *K* is the total number of potential adopters, and empirical constant *p* measures the effectiveness of available vertical communication channels. Mathematically, the model is equivalent to a differential equation

$$\begin{array}{c} \dot{N}=p\left(K-N\right) \end{array}$$

with a proper initial condition *N*(0) = *N*_0_ (the initial level of development). The diffusion curve, given by the solution to this equation, is characterized by a decaying exponential:

$$\begin{array}{c} N\left(t\right)=K(1-\left(1-N_{0}/K\right)\;e^{-pt}). \end{array}$$

The quantity *K* is usually called *carrying capacity*, because it is the maximum size that the technology can reach in the given conditions: *K* = lim_*t*→∞_ *N*(*t*).

The model of diffusion with purely internal influence was first proposed by Mansfield [24]. It is based on the assumption that there is a continuous interpersonal interaction among those already switched to a new technology and potential users of the innovation. Again, calling prior adopters *N* and adopters-to-be (*K* − *N*), the frequency of contacts between them at any instant of time would be proportional to *N*(*K* − *N*). This is mathematically similar to the law of mass action in chemistry by which the velocity of a chemical reaction is directly proportional to the product of the concentrations of the reactants. The respective differential equation takes the form

$$\begin{array}{c} \dot{N}=qN\left(1-N/K\right), \end{array}$$ (1)

where constant *q* is known as the coefficient of imitation. As a matter of fact, this is the famous *logistic equation* derived in 1838 by Verhulst [13] in the context of his studies in mathematical demography. Eq (1) has a solution

$$\begin{array}{c} N\left(t\right)=\frac{K}{1+\left(K/N_{0}-1\right)\;e^{-qt}}. \end{array}$$ (2)

Since its rediscovery in 1920 by Pearl and Reed [25], the logistic pattern of growth has been found in the fields as diverse as biology, ecology, medicine, economics, and sociology [11, 18, 26, 27]. Regarding to diffusion of technological innovations, the logistic equation, as a special case of epidemic model, has gained a wide popularity in modeling of *technological substitution*. By the latter is usually meant a process in which one product technology displaces another in performing a function (or functions) in an existing market. For binary processes—with two participating technologies—Fisher and Pry [28] developed a convenient transformation of the logistic function to a linear relationship. Namely, let *f* = *N*/*K* be the share of market served by the new technology at a given time *t*. Clearly, (1 − *f*) will be accounted for the traditional technology at the same instant. Then (2) may be represented in the form

$$\begin{array}{c} \text{ln}(\frac{f}{1-f})=a+bt, \end{array}$$

where *a* = ln(*N*_0_/(*K* − *N*_0_)) and *b* = *q*/*K*. Later on this model was extended and modified to describe the simultaneous development of multiple technologies [7].

The Bass model [14] contains both types of adopters: innovators and imitators. “Infection” by a novelty may occur both vertically (from external sources of information) and horizontally (via internal transmission of information). The differential equation of the model is as follows:

$$\begin{array}{c} \dot{N}=\left(p+qN/K\right)\left(K-N\right), \end{array}$$ (3)

where *p* and *q* are the respective empirical coefficients of innovation and imitation. On integrating (3), one obtains the growth formula

$$\begin{array}{c} N\left(t\right)=\frac{K(1+p/q)}{1+\frac{\left(K/N_{0}-1\right)e^{-(p+q)t}}{\left(K/N_{0}\right)\left(p/q\right)+1}}-K\left(p/q\right). \end{array}$$

The model proposed by Gompertz [15] in 1825 for the representation of actuarial data, has mostly been used to describe the development of body mass of certain tetrapods and the growth of populations of living cells (particularly tumors). Expressed in the form of a differential equation the model looks like

$$\begin{array}{c} \dot{N}=aN\;\text{ln}\left(K/N\right) \end{array}$$ (4)

with the associated integrated form being

$$\begin{array}{c} N\left(t\right)=K\;\text{exp}(-\text{ln}\left(K/N_{0}\right)\;e^{-at}). \end{array}$$ (5)

Mechanism underlying the Gompertz model is not an epidemic in a true sense. Its interpretation can be given in terms of the growth of a biomass *N* promoted by a growth factor *f* acting as a catalyst, whose dynamics is independent of the biomass. The growth factor is not consumed by the biomass, yet is not constant and breaks down gradually by itself. The corresponding system of equations is

$$\begin{array}{c} \dot{f}=-af,\;\dot{N}=bNf. \end{array}$$ (6)

It is an easy matter to check that system (6) is equivalent to Eq (4) with $K=N_{0}{\text{e}}^{bf_{0}/a}$, where *f*_0_ = *f*(0) and *N*_0_ = *N*(0).

Model (5) is widely used to fit empirical *S*-curves of technology diffusion. Leaving aside the pure regression-based works, the research by Kaldasch [29] seems noteworthy. The proposed analytical population-based model for market penetration of durable goods suggests that products play the role of species in biological evolution and each product can be characterized by its product fitness. The unit sales of products with a higher product fitness compared to the mean fitness increase, so these durables replace other goods. The model predicts in particular that the mean price exhibits an exponential decrease over a long time period for durable goods. The diffusion process is shown to be directly related to this price decline and therefore is governed by the Gompertz equation.

Although epidemic approach is not the only explanatory mathematical theory of technological diffusion, it is presently a *de facto* mainstream concept. The generic Bass Eq (3) is the simplest model that allows for broad intuitive interpretations and is consistent with empirical data. However, over the decades since its introduction, epidemic approach has attracted various criticisms owing to its weak economic background. We set off three aspects from a number of other seemingly questionable assumptions of epidemic models which severely limit their predictive power:

1) Market potential is supposed to remain constant [30];
2) The models have pro-innovation bias assuming that an innovation, once introduced to potential users, should be adopted. This bias denies the negative consequences of adopting a new technology. Only successful innovations are captured, while failures are not taken into account [31–33];
3) Spread of an innovation is not affected by diffusion of other innovations [30, 34]. In other words, epidemic models do not take into account competition between technologies, although competition is generally accepted as an essential component of a market economy. For many years, competition was thought to play a predominant role in structuring industries. Nowadays most economists choose to prefer a pluralistic view towards interactions. Nevertheless, there is no question of the overall importance of competition.

As we have mentioned, epidemic models are a part of more broad class of so-called population-based models. There is another approach within the framework of the population-based paradigm which attempts to address the issues listed above. The approach employs the famous *Lotka–Volterra–Gause* (*LVG*) competition equations borrowed from the mathematical ecology. It aims at describing the process of technologies substitution in terms of competition between technologies in much the same manner as competition between different living species.

Within a decade from the mid-1920s to the mid-1930s, Volterra [35], Lotka [36] and Gause [37] laid the foundations for the mathematical theory of competition by having proposed equations to model the dynamics of rival populations. In the representation brought by Volterra and Lotka interaction between two species feeding on the same resource is described by the system

$$\begin{array}{c} \begin{array}{cc} {\dot{N}}_{1} & =N_{1}(r_{1}-\gamma_{1}\left(h_{1}N_{1}+h_{2}N_{2}\right)),\\ {\dot{N}}_{2} & =N_{2}(r_{2}-\gamma_{2}\left(h_{1}N_{1}+h_{2}N_{2}\right)), \end{array} \end{array}$$ (7)

where *N*_*i*_ is the population of species *i* at time *t*, *r*_*i*_ is the intrinsic growth rate of the *i*th population. The equations assume that growth rate of *i*th population is effectively reduced by quantity *γ*_*i*_(*h*_1_ *N*_1_ + *h*_2_ *N*_2_), where (*h*_1_ *N*_1_ + *h*_2_ *N*_2_) is the total amount of food consumed by both species in a unit of time. The available food stock is decreased by *h*_*j*_ *N*_*j*_ through the action of *j*th species. It should be emphasized that the term (*h*_1_ *N*_1_ + *h*_2_ *N*_2_) does not depend on concrete species because both species consume the same food. However decrease of the food availability affects each species differently, so, in general, *γ*_1_ ≠ *γ*_2_. The coefficients *γ*_*i*_ and *h*_*i*_ are nonnegative. The model can be generalized to any number of competing species by introducing the intake function of a form ∑_*j*_ *h*_*j*_ *N*_*j*_.

An established biological fact is that closely related species, or species very similar in physical characteristics, habits, and feeding preferences, generally tend to compete more strongly when confined to the same habitat. In this connection, one may suppose the competing species in (7) are closely related, so that they rely on the same resource.

Consider the system (7) with *n* competitors. We can choose any pair of equations with numbers *k* and *l* and eliminate the term $\sum_{j=1}^{n}h_{j}N_{j}$ by multiplying the *k*th equation by *γ*_*l*_/*N*_*k*_ and the *l*th equation by *γ*_*k*_/*N*_*l*_, and then subtracting the former equation from the latter. As a result, we obtain

$$\begin{array}{c} N_{l}^{\gamma_{k}}/N_{k}^{\gamma_{l}}\propto \text{exp}((\gamma_{k}r_{l}-\gamma_{l}r_{k})t). \end{array}$$ (8)

If *r*_*k*_/*r*_*l*_ > *γ*_*k*_/*γ*_*l*_, then (8) yields lim_*t*→∞_ *N*_*l*_(*t*) = 0. Carrying out similar reasoning for each pair of populations from *n*, we find that after a long time, only one species will remain in the system. It is precisely the species with the greatest *r*/*γ* that survives. The case of *r*_*k*_/*γ*_*k*_ = *γ*_*l*_/*r*_*l*_, when two populations can coexist *ad infinitum*, is highly unlikely. A prediction of the Lotka–Volterra model of competition is that similar species in the same habitat will not coexist. This is a simplest version of the famous *principle of competitive exclusion* (e. g., [38]).

The competition model proposed by Gause is slightly more general as compared with (7). It has the form

$$\begin{array}{c} \begin{array}{cc} {\dot{N}}_{1} & =r_{1}N_{1}\left(K_{1}-N_{1}-a_{12}N_{2}\right)/K_{1},\\ {\dot{N}}_{2} & =r_{2}N_{2}\left(K_{2}-a_{21}N_{1}-N_{2}\right)/K_{2}, \end{array} \end{array}$$ (9)

where quantities *K*_1_ and *K*_2_ are the respective carrying capacities of species 1 and 2, when isolated, *a*_*ij*_ is the coefficient of competition expressing how much population *j* effectively reduces the growth rate of population *i*. By introducing an *n* × *n* interaction matrix (*a*_*ij*_) with all self-interacting (diagonal) terms *a*_*ii*_ ≡ 1, Eq (9) can be extended to *n* species competing against each other.

As it is easily seen, in a special case of *a*_12_ = 1/*a*_21_, Eq (9) are reduced to Eq (7) with *γ*_1_ = *r*_1_/*K*_1_, *γ*_2_ = *a*_21_ *r*_2_/*K*_2_, *h*_1_ = 1 and *h*_2_ = 1/*a*_21_. The Lotka–Volterra equations amended by Gause in the form (9) admit mechanisms of competition other than pure trophic, so the values *a*_12_ and *a*_21_ do not have to be equal.

The LVG Eq (9) take into account both *intraspecific* (between individuals of the same population) and *interspecific* (between members of different populations) competitions. These are expressed by respective negative quadratic terms $-N_{i}^{2}$ and −*N*_*i*_ *N*_*j*_. The model tells nothing about what the species are competing for nor does it tell anything about the way of competition. It merely postulates the net inhibitory effect of one population’s density on the own growth and on the growth of the counterpart.

The fixed points of system (9) are as follows:

$$N_{1}^{*}=N_{2}^{*}=0;$$ (10a)

$$N_{1}^{*}=K_{1},\;N_{2}^{*}=0;$$ (10b)

$$N_{1}^{*}=0,\;N_{2}^{*}=K_{2};$$ (10c)

$$N_{1}^{*}=\frac{K_{1}-a_{12}K_{2}}{1-a_{12}a_{21}},\;N_{2}^{*}=\frac{K_{2}-a_{21}K_{1}}{1-a_{12}a_{21}}.$$ (10d)

The trivial steady-state (10a) is where both species go extinct; the *boundary fixed points* (10b) and (10c) are where one species goes extinct and the other reaches its carrying capacity; and the *interior fixed point* (10d) lying in the first quadrant is the only steady state of coexistence.

A stability analysis of fixed points (10) reveals the conditions under which each species can survive:

1) Steady state (10a) is always unstable;
2) *a*_12_ < *K*_1_/*K*_2_ and *a*_21_ > *K*_2_/*K*_1_. Only (10b) is stable. Intraspecific competition is weaker than interspecific for species 1, and the other way round for species 2. Species 1 will always win out and drive species 2 to extinction;
3) *a*_12_ > *K*_1_/*K*_2_ and *a*_21_ < *K*_2_/*K*_1_. Only (10c) is stable. The situation is similar to the previous one, but the roles of species 1 and 2 are exchanged;
4) *a*_12_ < *K*_1_/*K*_2_ and *a*_21_ < *K*_2_/*K*_1_. Only (10d) is stable. For both populations intraspecific competition is stronger than interspecific. The species can coexist, although at a lower population than each by itself;
5) *a*_12_ > *K*_1_/*K*_2_ and *a*_21_ > *K*_2_/*K*_1_. Both (10b) and (10c) are stable, while (10d) is not. For both populations intraspecific competition is weaker than interspecific. Only one species can survive; which one depends on the initial conditions.

As one may see from the above, LVG competition model is capable of predicting not only a survival of the fittest, but also a coexistence of the rivals. The species having a higher carrying capacity always wins. Higher value of *K*_*i*_ means that given species can endure more crowding than the rival species.

Apparently, Batten [39] was first to apply LVG equations in the theory of technological diffusion. The new approach to diffusion was inspired by seminal contributions of Prigogine and his school at Brussels to the interdisciplinary studies on dissipative structures, complex systems, and irreversibility. A sluggish response to Batten’s paper from the *beau monde* of social scientists is due to common unpreparedness of that time. The outbreak of works [40–44], directed at the description of technology substitution in terms of LVG competition formalism, happened a decade later, in the 1990s, no sooner than the ideas of evolutionary economics became generally accepted.

For the sake of tractability, competing populations in the models of diffusion, based on LVG equations, are comprised of undifferentiated firms, each producing a single commodity utilizing a similar technology or set of resources. Each elemental firm in a population (industry) is thought to produce an output equal to the average output of the industry per firm. In this context, the definition for diffusion as “…the spread in the number of producers engaged in manufacturing a new product” by Gort and Klepper [45] seems more appropriate.

Taken overall, LVG-competition-based models in the forecited publications, as well as in the majority of more recent works [34, 46, 47], adequately mimic a broad range of diffusion patterns exhibited by accumulated empirical data and, at the same time, have far more profound economic content.

Meanwhile, since the late 1990s there is a strong tendency among researchers [43, 44, 48, 49] to go beyond solely competitive interactions and extend, in the spirit of evolutionary economics, the highly developed classification scheme of living species interactions to evolving technologies.

A major factor that can affect—hinder or promote—the spread of a technology in economy and society is its interactions with other technologies. Interactions between populations of firms can be classified as beneficial, detrimental, or neutral in their effects on each of the participating actors. Within the framework of the population models based on rate equations, the above interactions are respectively those increasing, decreasing, and having no impact on the (effective) growth rate of technologies.

Taking recourse to the generally accepted classification due to Odum [50, ch. 7], we can distinguish the following five essential types of pairwise interactions between technologies which rate equations could potentially model:

1) *Cooperation*. The type of relationship in which each actor benefits from the activity of the other.
2) *Competition*. This is the rivalry between actors for the most effective use of factors of production. There are two kinds of competition:
  a) In *direct interference*, the actors experience harm attributed to their mutual presence in a habitat (e. g., through aggressive behavior).
  b) In *exploitative*, or resource, competition, individuals and populations interact through utilizing (or occupying) a common resource that is in short supply (or abundance); the negative effect is due to the fact that one actor’s use of the resource reduces its availability to the other.
    The mode of competition is often determined by the nature of resource. If the resource is indivisible, the competition is that of interference, the fight will be direct. If the resource is divisible, the competition may be exploitative; the species will share the resource, accessing it at different times or places [51].
3) *Exploitation*. A collective term for a number of more specialized kinds of interactions, including consumer–resource, predator–prey, parasite–host, harvesting, fishing, and the like. In exploitation, one actor benefits at the expense of the other, regardless of trophic level. Between two trophic levels, exploitative interaction conventionally results in consumer–resource relations. Actors on the upper level, firms, consume factors of production from the lower level to transform them into products and services, and to reproduce themselves. At the same trophic level, however, i. e. between technologies, exploitation takes the specific form of *cannibalism*. Indeed, according to the epidemic models of technology substitution users of an old technology become adherents of a new technology without any costs for the latter. As this takes place, the new technology captures (cannibalizes) assets from the old one for free.
4) *Amensalism*. A type of interaction where one technology inflicts harm to the other without any costs or benefits received by itself. In economics terms, amensalism bears the meaning of the well-known negative externalities (negative spillover effects).
5) *Commensalism*. This interaction benefits one technology, while the other is neither benefited nor harmed. It occurs, for example, when a new technology takes benefits by recycling of wastes of another technology by which the host technology is not affected. Commensal relationships are also between *f* and *N* in Eq (6). Commensalism is equivalent to positive externalities.

Models of diffusion, where competition between technologies is treated in terms of the LVG equations, depict the phenomenon of competition without explicitly identifying an underlying mechanism. This is a major shortcoming of the approach under discussion. At first sight it would seem that classical LVG equations describe direct, or, in other words, interference, competition only, but not struggle for a common factor of production. It would be a hasty judgment though. In fact, LVG equations implicitly (in a parameterized form) do contain the resource competition also, however it is not possible to recover unambiguously what specific resource competition scheme the given LVG system corresponds to. This is because LVG equations actually are a result of adiabatic reduction of more complete system of so-called *consumer–resource* equations.

Consumer–resource equations are meant for modeling the interactions of exploitative type. They are also known as *predator–prey* equations. The very first predator–prey model was written by the founding fathers of mathematical ecology Lotka [52] and Volterra [35] in the form of two equations

$$\begin{array}{c} \begin{array}{cc} \dot{R} & =(a-bN)R,\\ \dot{N} & =(cbR-d)N. \end{array} \end{array}$$ (11)

Here *R* and *N* are the respective population densities of the resource (prey) and the consumer (predator); *a*, *b*, *c* and *d* are positive constant parameters. Resource is taken to be self-reproducible by a simple exponential law, *aR*, in the absence of consumer. The act of consumption (predation) only happens when two individuals physically meet. If the chance that, upon meeting a prey, the predator will eventually eat it, is given by *b*, then the total prey consumed in a unit of time is therefore *bNR*. Some fraction of the biomass of an eaten prey is transformed into predator biomass, so the growth of the predator population equals *cbNR*, where *c* is the yield coefficient telling how many units of predator are produced from the consumption of one unit of prey (conversion efficiency of prey biomass into predator biomass). Also, predators are assumed to die naturally with the frequency *d*. System (11) is generic for all models aimed at description of the resource competition.

MacArthur [53] was first to derive LVG equations from consumer–resource equations for *n* species feeding on *m* resources of a reasonably general form

$${\dot{R}}_{l}=(b_{l}\left(1-R_{l}/k_{l}\right)-\sum_{i=1}^{n}c_{il}N_{i})R_{l},\;l=1,\ldots ,m,$$ (12a)

$${\dot{N}}_{i}=(\sum_{l=1}^{m}w_{il}c_{il}R_{l}-d_{i})N_{i},\;i=1,\ldots ,n.$$ (12b)

The resources in (12a) are assumed to be *biotic* (self-reproducible) featuring a logistic population growth. Here *R*_*l*_ represents the total biomass of the *j*th resource (prey), *N*_*i*_ stands for the total biomass of the *i*th consumer (predator), *b*_*l*_ and *k*_*l*_ are the respective growth rate and carrying capacity of the *l*th resource, *c*_*il*_ is the rate of uptake of a unit of *l*th resource by each individual of the *i*th consumer population, *w*_*il*_ is the conversion efficiency of the *i*th consumer regarding the *l*th resource, *d*_*i*_ is the loss rate of the *i*th consumer’s biomass due to natural death, migration and physiological maintenance. All parameters in (12) are nonnegative.

MacArthur assumed the population dynamics of the resources to be much faster as compared to that of the consumers. As a consequence, *R*_*l*_ in (12b) can be approximated by its steady-state value derived by setting the right-hand side of Eq (12a) to zero. The validity of such a procedure, also called *adiabatic elimination*, may be justified by application of the Tikhonov theorem known from singular perturbations analysis (e. g., [54, ch. 8]). With this substitution, “slow” Eq (12b) takes the standard LVG form

$$\begin{array}{c} {\dot{N}}_{i}=(r_{i}-\sum_{j=1}^{n}a_{ij}N_{j})N_{i},\;i=1,\ldots ,n, \end{array}$$

where $r_{i}=\sum_{l=1}^{m}c_{il}k_{l}w_{il}-d_{i}$ and $a_{ij}=\sum_{l=1}^{m}c_{il}c_{jl}k_{l}w_{il}/b_{l}$.

Clearly different resource-partitioning schemes may lead to an LVG model with the same parameters. Parametrization of trophic competition through coefficients of direct competition can be carried out for an *abiotic* (non-self-reproducible) resource as well. For instance, an adiabatic reduction has been applied to an open system where species (with continuous trait) consume the common resource that is constantly supplied, under the assumption of a very fast dynamics for the supply of the resource and a fast dynamics for death and uptake rates [55]. Is, however, the very supposition of relative rapidity of the resource variable justified in all situations? In biological communities, the most common case is, indeed, rapid consumption of food by species. But even in ecological systems, if we consider the first level, at which the consumers are autotrophs and the resources are mineral nutrients, the environmental conditions may be quite stable on the evolutionary timescale, the inflows of inorganic substrates from the surroundings may be considered constant and the washout time of a substrate may occur much longer than the life expectancy of a species. A model of two consumer–resource pairs linked by direct competition of consumers has been suggested [56] with the basic assumption of a relative slowness of the resource. The model demonstrates modes of behavior qualitatively different from those if reduced to an LVG system under conventional assumptions.

From the economics perspective, the slow–fast dichotomy between technologies (as consumers) and utilized resources is by no means univocal. A characteristic example thereof is rendered by an attempt of Brander and Taylor [57] to reconstruct the economics of *Rapa Nui* (Easter Island) with a plausible predator–prey model. The model suggests that the slow dynamics of the principal resource, palm forest, relative to the span of an individual human life would cause non-monotonic, oscillatory, convergence toward the steady-state population.

Attempts to link competition coefficients to the abundance and diversity of resources lead to necessity of taking into account the dynamics of the latter. Models of technological diffusion based on LVG equations are lacking the very object of competition—factors of production. Instead, a vague notion of the carrying capacity is in use. At the same time, it is well known that resource supply imposes crucial constrains on both diversity of producers and their number. Virtually the entire *industrial ecosystem*, as termed by Frosch and Gallopoulos [58], is closely tied to the techno-economic characteristics of resource yield and consumption. Technological structure and functioning of an economy depend ultimately on the availability of resources. Quantitative characteristics of external limiting resources, the regulators of technological structure, are not contained in LVG equations written only in terms of populations of firms (or their outputs). Any plausible explanatory population-based model ought to give clear answers to the following questions about the conditions of coexistence of competitors or winning of one of them:

(i) Suppose several technologies are in a competitive relationship; will one of them necessarily drive others to complete extinction?
(ii) Vice versa, does a long-term coexistence of technologies mean, that there is no competition between them?

Within the framework of the diffusion models based on LVG equations, only intuitive answers to these questions are obtainable due to the abstractness of the carrying capacity concept. Thus, to develop a consistent evolutionary theory of technological change compliant with practical challenges of innovation policy, new models of interacting populations of firms built on more intelligible economic basis are needed.

Construction of the models for mechanisms of technology coexistence and regulators of the technological structure in economic industries of varying degrees of complexity is imperative. A meaningful theory is to be able to provide answers (or clues) to such important, in practical terms, questions as:

(i) What are the criteria for a technology to win and become dominant?
(ii) To what extent can technologies be diverse and abundant in a given economic environment?
(iii) What are the necessary conditions for a long-term coexistence of a certain set of technologies?
(iv) Why are some technologies more abundant and the others less?
(v) What structural changes will be brought about by introduction (elimination) of a certain group of technologies to (from) an industry?

The ability to answer these questions could expand the possibilities of forecasting the impact of a given innovation on economic development, which is necessary for implementation of effective technological policy.

The purpose of the present work is to build and investigate a mathematical model of the evolution of technological structure of an economic system, consisting of populations of firms that compete with each other through the consumption of essential resources. In accordance with the assigned objective, the following tasks are being solved:

1) To formulate general consumer–resource equations for an open system wherein different technologies mutually interact via consumption of essential resources;
2) To find out how input and output of an elemental firm depend on the resource availability under the realistic assumptions about firm’s supply chain;
3) To formalize the notion of competition outcome as applied to technologies in terms of dynamical systems;
4) To identify the criterion of technology’s efficiency;
5) To ascertain the role of resource diversity in an established technological structure of the system;
6) To formulate conditions for compatibility of technologies.

## The model

As is the convention in most economic taxonomies, similar companies are grouped together into industries based on the primary product/service that a company produces or sells. (In turn, industries are grouped together into sectors.) Suppose that there are *n* different technologies in a certain industry. Let *N*_*j*_ be a measure of the spread of *j*th technology, where *j* = 1, …, *n*. Following Batten [39], we assume *N*_*j*_ to be the population of weakly differentiated, elemental, firms utilizing given technology *j* to produce a similar commodity with like average output. Suppose there are *m* distinct resources each of *n* technologies consumes and ultimately converts to its final product. Let *R*_*i*_ be the total stock of *i*th resource (*i* = 1, …, *m*) available for all *n* technologies in the system at instant *t*. Our point of departure is the following system of consumer–resource equations:

$$\begin{array}{c} \begin{array}{cc} {\dot{R}}_{i} & =r_{i}-\sum_{j=1}^{n}N_{j}Q_{ij}Y_{j}-d_{i}R_{i},\;i=1,\ldots ,m,\\ {\dot{N}}_{j} & =N_{j}(h_{j}Y_{j}-D_{j}),\;j=1,\ldots ,n. \end{array} \end{array}$$ (13)

Here overdots indicate differentiation with respect to time *t*, *r*_*i*_ defines the rate with which *i*th resource is injected to the industry, *Q*_*ij*_ is the content of resource *i* in the product of type *j*, *Y*_*j*_(⋅) is the product yield of an individual firm of *j*th type (the dot indicates *Y* may depend on various quantities, including possibly *R* and *N*), *d*_*i*_ is the per unit outflow rate (depreciation) of *i*th resource, *h*_*j*_ is the conversion factor showing how many new firms of *j*th type could be set up on proceeds from one unit of *j*th product, and *D*_*j*_ is firms’ exit rate from the population, i. e. the per firm loss rate by *j*th technology due to all forms of loss including closures, maintenance expenses and the like.

Eq (13) are inspired by the model of *chemostat*—cultivation bioreactor that feeds nutrients at a constant rate to a population of microorganisms in a fixed volume [59].

According to nomenclature coined by Solomon [60] and generally accepted in ecology, terms *Y*_*j*_ and *h*_*j*_ *Y*_*j*_ are the respective *functional* and *numerical* responses of *j*th consumer/predator to a given set of resources/preys. Functional response is also referred to as the *trophic function*. We assume that the two responses differ by a constant factor.

Economically, *Y*_*j*_ is the *production function* of a firm of *j*th type. By definition, the production function yields a per firm production rate (*output*) obtainable with the specific technology from a given set of *factors of production*. It is appropriate to say a few clarifying words about the terms used in what follows. We restrict ourselves to considering the physical factors of production. In the broad sense of the word, a factor of production is any entity, the degree of availability of which positively correlates with the output. Factors of production are of two kinds: consumable and nonconsumable. It is convenient to refer to consumable factors as *resources*, as it is generally accepted in ecology, despite the fact that economists often do not distinguish between resources and factors.

The consumption of a material resource is always aimed at reducing its availability. As the production process takes place, the resource is consumed, used up. By consumption we understand irreversible conversion, physical embodiment, of a resource into a material product.

Nonconsumable factors of production are referred to as *funds*. They are not resources in the sense of the above definition, but this fact in no way diminishes their importance. Funds are not materially transformed into an output they produce. They are transforming tools that turn the involved resources into a product, but are not themselves embodied physically in the product. Although funds are not used up, their amount can change and they are subject to wear-and-tear. Common examples of funds are capital, labor, and land.

These are examples of what we would say is ability of funds to vary: machines in a production line can be switched on or off at the direction of a factory manager; some workers can be transferred from one site to another in case of necessity; a farmer may decide a part of the arable land to be left uncultivated or used for service areas (housing, buildings, etc.). However, such on-the-run adjustments being responses to abrupt changes in external environment, where a given business operates, happen rather infrequently. Most of the time, funds are maintained fixed to comply with the nominal production output. On the other hand, funds also may wear out: equipment and machinery either fail or become obsolete, workers get tired, agricultural land degrades over time. However, factories usually practice various planned replacement, repair and maintenance programs, employees always work in shifts and also are entitled to a periodic leave, and farmers try to leave a certain percent of their arable land fallow. Thanks to the above measures, amount of fund factors never declines. Thus, assuming normal operation of a firm for most of its life, funds may be considered constant for modeling purposes.

Dimensionally speaking, production function is a flow quantity. Its resource-related arguments are usually called *inputs* for short. Most commonly they are flows, but also may be stocks, as it is the practice in industries like agriculture, forestry and logging, and fishery. The first case deals with the relation between resource supply rate and production rate, while the second with the relation between resource availability and production rate. Arguments from the category of funds always enter into the production function as stock variables.

**Fig 1:**
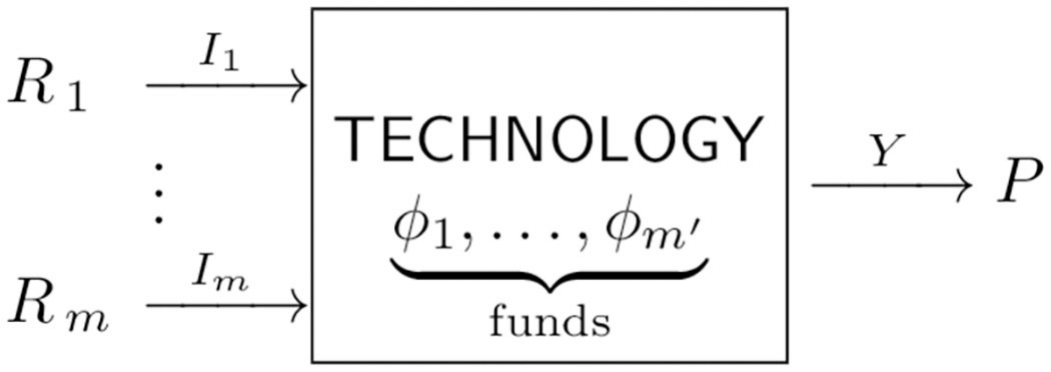
Representing technology as a converter of material resources into product. The set of resources *R*_1_, …, *R*_*m*_, which can be raw materials, semiproducts, parts, or other commodities, are jointly transformed into a product *P* under the “catalytic” action of the set of funds *ϕ*_1_, …, *ϕ*_*m*′_. The arrow labels stand for respective flows.

Any manufacturing technology may be conceived as a converter of a set of resources into a product by means of the funds (Fig 1). Resources are fed to the converter from the outside, while funds act inside of the black box of the technology. Funds are not spent on the output, however they function to make the transformation of resources to product feasible and efficient, and to enable control of that transformation.

Schumpeter [61, p. 87] identified innovation with “…the setting up of a new production function”. When applied to technology as unique manufacturing method, production function is expected to not allow substitutability between the consumed resources. To formalize technology, Leontief [62, p. 38] postulated a simplest linear fixed-proportions form of production function named for its originator. In view of the above, the appropriate choice for *Y*_*j*_(⋅) would be the Leontief production function

$$\begin{array}{c} Y_{j}=\text{min}(I_{1j}/I_{1j}^{0},\ldots ,I_{mj}/I_{mj}^{0}), \end{array}$$ (14)

where *I*_1*j*_, …, *I*_*mj*_ are the inputs, and $I_{1j}^{0},\ldots ,I_{mj}^{0}$ are the technical (“stoichiometric”) coefficients measuring the input requirements per unit of *j*th output.

Production function (14) is in the flow representation, however it should be in the stock form to make model (13) self-contained.

Georgescu-Roegen [63] and, subsequently, Poletaev [64] and Chernavskii [65, p. 134], put forward an idea that, in terms of cybernetics, funds act by the same mechanism as catalysts and enzymes. Indeed, in a living cell, a substrate molecule, *S*, binds to an enzyme, *E*, to form a short-lived substrate-enzyme complex, [*ES*]. The complex then breaks up into a product, *P*, and the original enzyme, which can then catalyze a new reaction (e. g. [66]):

$$\begin{array}{c} S+E\to [ES]\to P+E. \end{array}$$ (15)

The role of enzyme concentration is played by the number of machines in a shop or automatic line. Based on this idea, it can be shown (ref. for the details [67]) that virtually any converging, multi-resource, single-product supply chain of arbitrary length must possess the property of Leontief technology: its output will be solely determined by the slowest input. Assuming the input (upstream) stage corresponding to *i*th resource to operate by the generic mechanism (15), an uptake rate of *i*th resource will be described by the famous *Michaelis–-Menten equation* [68] for substrate uptake rate in enzymatic reaction:

$$\begin{array}{c} I_{ij}=\frac{{\hat{I}}_{ij}S_{i}}{K_{ij}+S_{i}}. \end{array}$$ (16)

Here *S*_*i*_ is the *per firm availability* of *i*th resource, ${\hat{I}}_{ij}$ is the maximum uptake rate of *i*th resource by a firm of *j*th type, and *K*_*ij*_ is the *half-saturation constant*—the value of *S*_*i*_ when $I_{ij}=\frac{1}{2}{\hat{I}}_{ij}$, i. e. the resource stock supporting an uptake rate one-half the maximum rate. It must be emphasized once more that *S*_*i*_ in (16) has different physical meaning as opposed to the absolute substrate concentration in the Michaelis–Menten equation. At level of firm, (16) is also identical to the *Monod equation* for the rate of substrate utilization by microorganisms in a nutrient solution [69] and the *Holling’s type-II functional response* of predator to prey population density [70].

Substituting (16) in (14) we obtain the sought-for stock representation for the Leontief production function:

$$\begin{array}{c} Y_{j}=\text{min}(\frac{{\hat{Y}}_{1j}S_{1}}{K_{1j}+S_{1}},\ldots ,\frac{{\hat{Y}}_{mj}S_{m}}{K_{mj}+S_{m}}), \end{array}$$ (17)

where ${\hat{Y}}_{ij}={\hat{I}}_{ij}/I_{ij}^{0}$.

As a matter of fact, Eq (17) is a mathematical version of the famous *law of the minimum* commonly credited to a German chemist Liebig [71]. This law states that the rate of growth of a plant or crop is affected not by the most abundant mineral resource, but by the most deficient one. Essentially, a plant will only yield as much as the least available nutrient allows. So, more correctly (17) should be called the *Leontief–Liebig production function*.

Plugging (17) in (13), introducing the quota of *i*th resource in a firm, *q*_*ij*_ = *Q*_*ij*_/*h*_*j*_, and the maximum per firm growth rate of *j*th technology due to *i*th resource, $\gamma_{ij}=h_{j}{\hat{Y}}_{ij}$, and taking into account that $S_{i}=R_{i}/\sum_{j=1}^{n}N_{j}$, we get model equations in the following explicit form:

$${\dot{R}}_{i}=r_{i}-\sum_{j=1}^{n}q_{ij}N_{j}G_{j}-d_{i}R_{i},\;i=1,\ldots ,m;$$ (18a)

$${\dot{N}}_{j}=N_{j}\left(G_{j}-D_{j}\right),\;j=1,\ldots ,n;$$ (18b)

$$G_{j}=\text{min}(\frac{\gamma_{1j}R_{1}}{R_{1}+K_{1j}N_{\Sigma }},\ldots ,\frac{\gamma_{mj}R_{m}}{R_{m}+K_{mj}N_{\Sigma }}),$$

$$N_{\Sigma }=\sum_{j=1}^{n}N_{j}.$$

Eq (18) belong to a type of *ratio-dependent* predator–prey models according to the term coined by Arditi and Ginzburg [72], strong advocates of this approach to the functional response since the 1970s. We believe, for population of firms the ratio-dependent alternative is more appropriate compared with the traditional Lotka–Volterra (as well as the Monod–Holling) strictly prey-dependent description. Intra-technological competition among closely related firms for essential resources does exist in economic life and it becomes more pronounced as the technology grows up in a given industry. Mechanistically, this competition could be taken into account by appending a quadratic term $c_{j}N_{j}^{2}$ to the right-hand side of (18b). The quadratic term (first introduced by Verhulst [13]) assumes that the inhibitory effect of crowding on the population growth under conditions of limited resources is proportional to the number of contacts (encounters) between individual members of population. In spite of simplicity, such an interpretation does not point out explicitly to the object of competition. It rather describes a direct (interference) kind of competition, than the resource-based (trophic) form. Our model, as is evident from the structure of Eq (18), explicitly accounts for the intra-technological competition arguing that the rate of consumption of a certain resource by an individual firm is proportional to the number of units of that resource available to the firm, rather than to the total stock of the resource in the industry or economy.

All parameters in the above equations are nonnegative. One can readily note that, in essence, the Leontief term in the right-hand side of Eq (18b) is the per firm entry rate of new firms to population *j* with maximum value *γ*_*ij*_. The half-saturation constant *K*_*ij*_ is inverse measure of the affinity of *j*th technology to *i*th type of resource. Coefficients *γ*_*ij*_ and *K*_*ij*_ are hereditary traits of *j*th technology *per se* because both determine the unique style of production. Quota *q*_*ij*_ is the technical coefficient determining the *i*th resource requirements per one new firm of *j*th type. A lower *q*_*ij*_ indicates that less resource *i* is needed to set up another firm in *j*th population on proceeds from the production output.

As to parameters *r*_*i*_, *d*_*i*_ and *D*_*j*_, they characterize an economic environment where the technology grows.

Dimensions of all quantities involved in Eq (18) are given below:

$$\begin{array}{cccc} \left[R_{i}\right] & =\text{units}\;\text{of}\;\text{resource}, & \left[N_{j}\right] & =\text{numbers}\;\text{or}\;\text{firms},\\ {}[r_{i}] & =\frac{\text{units}\;\text{of}\;\text{resource}}{\text{units}\;\text{of}\;\text{time}}, & \left[q_{ij}\right] & =\frac{\text{units}\;\text{of}\;\text{resource}}{\text{firm}},\\ {}[K_{ij}] & =\frac{\text{units}\;\text{of}\;\text{resource}}{\text{firm}}, & \left[\gamma_{ij}\right] & =\frac{1}{\text{units}\;\text{of}\;\text{time}},\\ {}[d_{i}] & =\frac{1}{\text{units}\;\text{of}\;\text{time}}, & \left[D_{j}\right] & =\frac{1}{\text{units}\;\text{of}\;\text{time}}. \end{array}$$

Thus system (18) describes *n* technologies feeding on *m* nonsubstitutable resources. By the analogy with biocenosis—a closely integrated association of different species in a given biotope—the community of dynamically interacting technologies may be called *technocenosis*.

## Analysis and implications

### An isolated technology

Consider the growth of a new technology in the absence of interactions with incumbents. This dynamics is described by Eq (18) with *n* = 1. Suppose we are able to distinguish some resource that is in the shortest supply all the time providing all other factors of production are in abundance. Under these circumstances we may drop all but one of the equations for resources in (18) resulting in the system

$$\dot{R}=r-\frac{q\gamma RN}{R+KN}-dR,$$ (19a)

$$\dot{N}=\frac{\gamma RN}{R+KN}-DN.$$ (19b)

Eq (19) will be studied for two different cases: closed and open technology–resource system.

#### Closed system

We start with the simple case of a closed system, where the limiting resource of a certain finite initial stock neither arrives nor goes to waste, and firms never leave the population, so *r*, *d*, *D* = 0. To gain better insight into properties of the system, we will pass on to new dimensionless quantities $x=R/(q\tilde{N})$, $y=N/\tilde{N}$, *τ* = *tγ*, and *ϰ* = *K*/*q*. The normalization constant $\tilde{N}$ may be chosen at will. Making these substitutions into (19) leads to a nondimensional system

$$\begin{array}{c} \dot{x}=-\frac{xy}{x+\varkappa y},\;\dot{y}=\frac{xy}{x+\varkappa y}, \end{array}$$ (20)

where overdots now denote differentiation with respect to *τ*. We will imply that the system is supplemented with suitable initial conditions *x*(0) = *x*_0_ and *y*(0) = *y*_0_.

System (20) has obvious first integral *x* + *y* = *x*_0_ + *y*_0_ meaning that the sum *x* + *y* remains constant on solution curves (*x*(*τ*), *y*(*τ*)). Reducing (20) to a single equation for *y*, we get

$$\begin{array}{c} \dot{y}=\frac{y(x_{0}+y_{0}-y)}{x_{0}+y_{0}+\left(\varkappa -1\right)y}. \end{array}$$ (21)

Although there is no explicit formula for the solution of (21) in the form *y*(*τ*), one can readily write down the solution as a relationship between *τ* and *y*:

$$\begin{array}{c} \text{ln}(\frac{y}{y_{0}})+\varkappa \;\text{ln}(\frac{x_{0}}{x_{0}+y_{0}-y})=\tau . \end{array}$$ (22)

**Fig 2:**
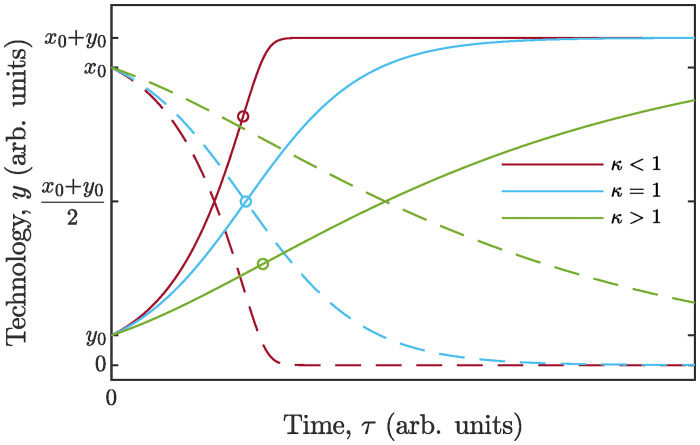
Diffusion of an isolated technology feeding on an exhaustible limiting resource. The nondimensional variables are used (ref. (20)). Solid *S*-curves depict time profiles of population of firms, dashed lines represent kinetics of resource. The position of an inflexion point (indicated by a circle) on concrete *S*-curve is determined by the value of the parameter *ϰ* of the model. In particular, *ϰ* = 1 yields the logistic growth law.

This formula predicts that, in general terms, the time profile of the technology will be a kind of *S*-curve (Fig 2). Moreover, as the technology grows, the resource is depleted and eventually gets exhausted. It is seen from (22), that the technology grows exponentially as long as the resource is abundant. As the time goes on, the resource becomes scarce and the number of firms tends to the saturation value (*x*_0_ + *y*_0_), which plays the role of a carrying capacity. In the special case of *ϰ* = 1, Eq (22) degenerates into a logistic function.

**Fig 3:**
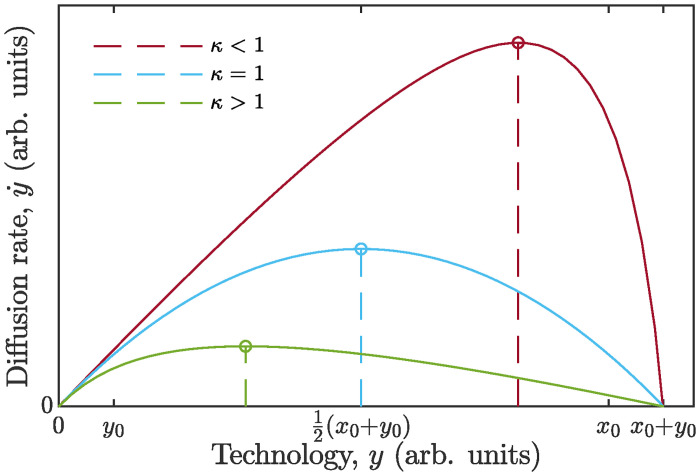
Diffusion curves, generated by model (21), in the plane $\left(y,\dot{y}\right)$. The value of *y*, which delivers zero to $d\dot{y}/dy$, corresponds to the inflection point (indicated by dashed line) in the domain of admissible values. The curves are right-skewed for *ϰ* > 1, and left-skewed for *ϰ* < 1. The case *ϰ* = 1 features a logistic growth curve.

The study of Eq (21) in the plane $\left(y,\dot{y}\right)$, as presented in Fig 3, allows us, among other things, to predict the position of the point of inflection on *S*-curve depending on the parameter *ϰ*. The inflection point corresponds to a maximum of the function (21), that is to the maximum growth rate of the technology. Namely, ${\dot{y}}_{\text{max}}=\left(x_{0}+y_{0}\right)/{\left(1+\sqrt{\varkappa }\right)}^{2}$ occurs at $y_{\text{infl}}=\left(x_{0}+y_{0}\right)/\left(1+\sqrt{\varkappa }\right)$. One may see that for *ϰ* > 1 growth rate peak will be attained at a population size below one half of the carrying capacity, whereas for *ϰ* < 1 the peak will happen at a level of development above one half of the maximum. Analyzing all three basic *S*-curves we find that the Verhulst (logistic) model (1) yields inflection point exactly at $N=\frac{1}{2}K$, the Bass model (3) at $N=\frac{1}{2}K\left(1-p/q\right)<\frac{1}{2}K$, and the Gompertz model (4) at $N=K/e<\frac{1}{2}K$, where *K* is the carrying capacity. All the above cases can be covered by Eq (22) by proper adjusting the value of *ϰ*. Thus, the proposed new model (20) for diffusion of an isolated technology with exhaustible limiting resource combines simplicity and sufficient flexibility.

As we have seen, according to our model, if the resource is nonrenewable, the diffusion of a technology will follow an *S*-curve given by Eq (22). The reverse statement can also be made: the observed *S*-shaped growth could be a sign of the depletion of some limiting resource. This does not contradict to the mainstream epidemic models of technology diffusion. Indeed, the subpopulation of potential adopters may be regarded as an exhaustible resource of a sort from the perspective of our model. More precisely, it is not the potential adopters themselves, but their solvent demand for the novelty that acts as a limiting resource.

**Fig 4:**
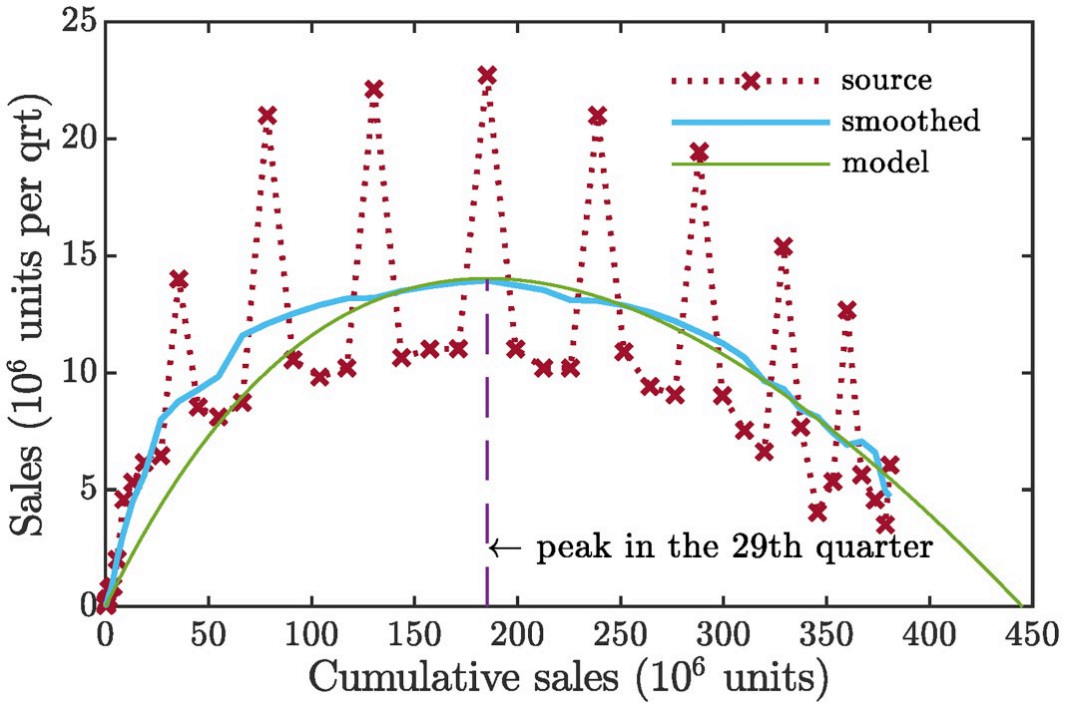
Apple’s iPod penetration chart (2002–2014) in the context of the model (21). A raw time series, ${\dot{y}}_{i}\;\left(i=1,\ldots ,49\right)$, representing quarterly sales of iPod (in million units) over a period covering 49 consecutive quarters can be found on website [73]. It is the following set of figures: {0.125, 0.057, 0.054, 0.14, 0.219, 0.078, 0.304, 0.336, 0.733, 0.807, 0.86, 2.016, 4.58, 5.311, 6.155, 6.451, 14, 8.526 8.111, 8.729, 21, 10.549, 9.815, 10.2, 22.121, 10.644, 11.011, 11.022, 22.722, 11.01, 10.2, 10.2, 21, 10.89, 9.41, 9.051, 19.45, 9.02, 7.535, 6.62, 15.397, 7.673, 4.02, 5.344, 12.679, 5.633, 4.569, 3.498, 6.049}. This series first was subject to smoothing by taking the moving average over a four-quarter sliding window, and then fitted by the Eq (21).

As a numerical example, we consider an *S*-curve of the iPod, Apple’s pocket-sized music device regarded as one of the company’s biggest successes. Quarterly data for worldwide unit sales have been obtained from AAPLinvestors [73]. For the iPod, the data embrace 49 consecutive quarters beginning from Q1 2002, when the product was first introduced, and extending to the fiscal Q1 2014, when Apple stopped breaking out iPod sales separately in its earnings reports. (The company’s fiscal year starts in October). To dampen seasonal fluctuations, both quarterly data and cumulative data have been smoothed out prior to use by means of the moving average procedure. The resulting graph for quarterly sales versus cumulative sales is presented in Fig 4. The product’s lifecycle peaked in Q1 2009, when the cumulative sales totaled 185 million units. This is just the inflection point. Our model can be fitted to the averaged empirical data assuming the growth potential for the product to be 445 million customers and *ϰ* = 1.98. The example is not intended to demonstrate an advantage of our model as a forecasting tool compared to the mainstream models of diffusion, but rather to illustrate workability of the theoretical concept.

#### Open system

Now turn back to Eq (19) for the general case of continuous-flow resource and nonzero exit rate. Given the constant resource supply, growing number of the consumers will lead to shrinkage of resource consumption per one firm (*scramble competition* in terms of ecology). This will entail decline of per firm output and cutback of investment in setting up new firms. Such a negative feedback provides the existence of a dynamic equilibrium between the number of firms and the current stock of available resource. The steady-state growth limit of the technology is established as a result of the intra-technological competition (between firms sharing the same technology) for the common limiting resource.

System (19) has two fixed points:

$$\begin{array}{cc} F_{1}:\;\bar{R}=r/d, & \;\bar{N}=0; \end{array}$$ (23a)

$$\begin{array}{cc} F_{2}:\;\bar{R}=\frac{Kr}{dK+q(\gamma -D)}, & \;\bar{N}=\frac{r(\gamma -D)}{D(dK+q(\gamma -D))}. \end{array}$$ (23b)

**Fig 5:**
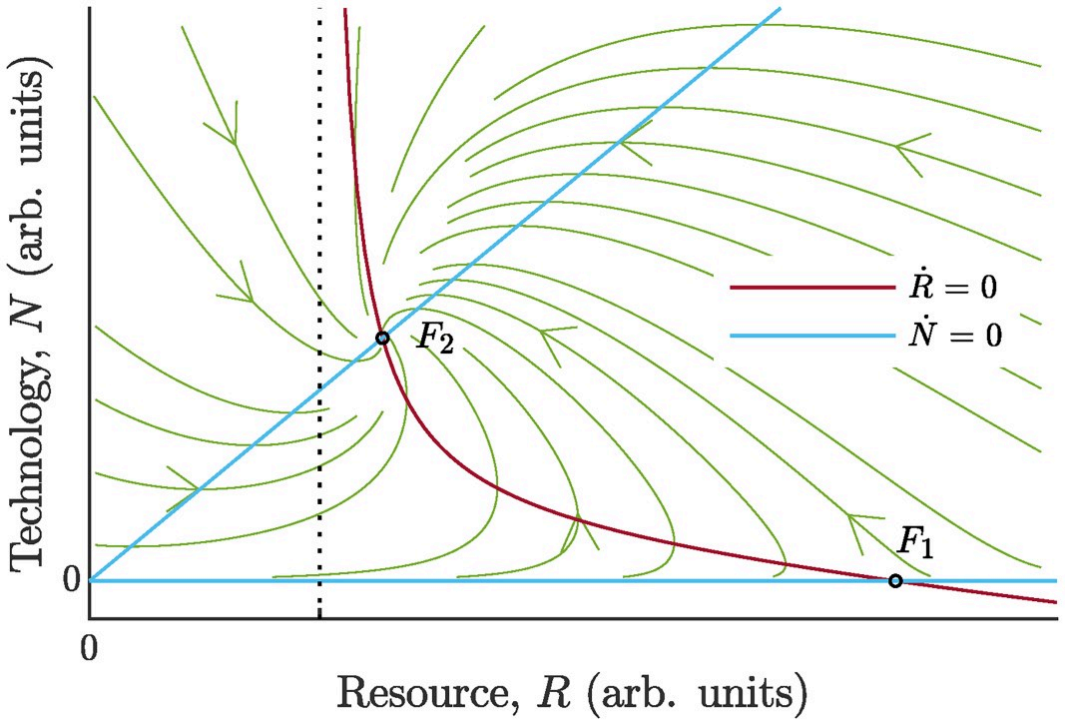
Phase portrait of the system (19). The unpopulated steady state *F*_1_, given by (23a), is a saddle point, while the positive equilibrium, *F*_2_, given by (23b), is a stable node. The steady-states lie on the intersections of the nullclines. The vertical nullcline $\dot{R}=0$ is the hyperbola *N* = *R*(*r* − *dR*)/((*dK* + *γq*)*R* − *Kr*) with the asymptote *R* = *Kr*/(*dK* + *γq*) (shown by the dotted line). The horizontal nullcline $\dot{N}=0$ consists of two lines, *N* = 0 and *N* = *R*(*γ* − *D*)/(*DK*).

The phase portrait of Eq (19) is presented in Fig 5. The unpopulated steady state (23a) corresponds to “washout” of the technology from the system; it always exists. Steady state (23b) with physically meaningful positive number of firms may take place only for *D* < *γ*. Indeed, the case *D* > *γ* leads to $\dot{N}<0$ for all values of *R* and, as a result, exit rate among the firms would surpass entry rate. Hence, *D* < *γ* is the feasibility condition for (23b).

**Fig 6:**
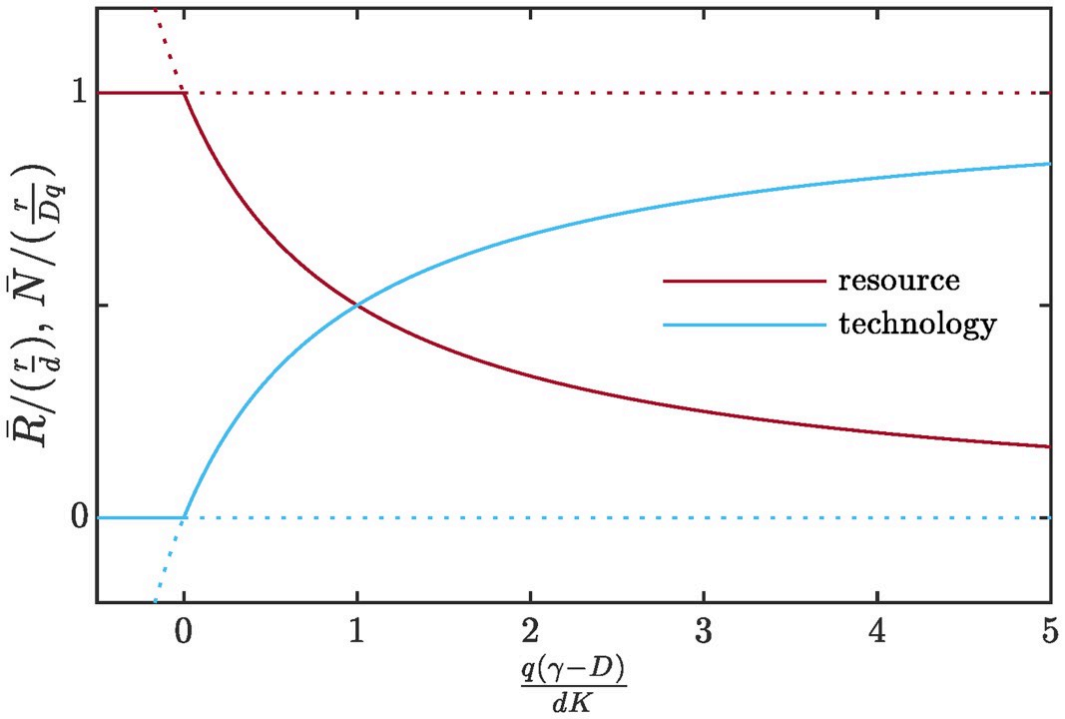
Bifurcation diagram for system (19). The solid lines depict stable behavior and the dotted lines depict unstable behavior. For *D* < *γ*, there is an unstable fixed point *F*_1_ (ref. Eq (23a)) and a stable fixed point *F*_2_ (ref. Eq (23b)). As *D* increases toward *γ*, fixed point *F*_2_ approaches fixed point *F*_1_, and coalesces with it when *D* = *γ*. Finally, when *D* > *γ*, fixed point *F*_2_ has become unstable, and fixed point *F*_1_ is now stable. There is an exchange of stabilities between the two fixed points.

Depending on the ratio *γ*/*D*, either of fixed points (23) may prove to be stable node or saddle. The two steady states are never both stable together. They coalesce and exchange stabilities via a *transcritical bifurcation* at *D* = *γ* in the manner indicated in Fig 6. Nontrivial steady state (23b) is always stable whenever feasible, i. e. at 0 < *D* < *γ*, whereas fixed point (23a) is stable at *D* > *γ*.

In steady state, the amount of resource that falls to an individual firm’s share is given by

$$\begin{array}{c} S^{*}=\frac{K}{\gamma /D-1}. \end{array}$$ (24)

By analogy with chemostat, this may be called the *break-even resource availability* at which entry rate equals exit rate (e. g., [74, p. 9]). (If *D* ⩾ *γ*, the convention is to set S* = + ∞.) It represents per firm quantity of resource that the given technology requires in order to maintain a stable equilibrium level. At a given exit rate (dependent on a specific business environment) the break-even level is a unique performance characteristic of the technology.

Illustration of model (19) with empirical data is not quite a straightforward task. The conventional representation for the Leontief production function is flow-based relation between resource supply rate and production rate. As a notable exception, a few suitable data sets were found in agriculture. Agricultural economists sometimes employ stock-based production functions giving the relation between soil nutrient concentration and crop at the root level.

Nijland et al. [75] argued that crop production of such culture as feed grass as a function of two nutrient inputs, nitrogen and water, may be described adequately by a two-factor production function in stock representation. To put it precisely, the production function they fitted to available empirical data was not exactly a fixed proportions production function (17) used in our model. Their response function has the reciprocal form

$$\begin{array}{c} Y^{-1}={\hat{Y}}^{-1}+{\left(a_{n}S_{n}\right)}^{-1}+{\left(a_{w}S_{w}\right)}^{-1}+{\left(a_{nw}S_{n}S_{w}\right)}^{-1}, \end{array}$$ (25)

where *Y* is the rate of dry matter production, $\hat{Y}$ is a climatically determined maximum (called the potential production rate), *S*_*n*_ and *S*_*w*_ are the respective concentrations of nitrogen and water in soil, *a*_*n*_ and *a*_*w*_ are the affinity coefficients for nitrogen and water, respectively, defined as the production rate per unit nutrient when nutrient is almost zero, *a*_*nw*_ is a measure for the extra response of production to the combination of nitrogen and water—apart from the separate responses *a*_*n*_ and *a*_*w*_ to these nutrients.

Assuming nitrogen is in abundance, Eq (25) is reduced to a production function just for water. The work being cited brings the following water-related figures concerning grass: $\hat{Y}=20\;\text{ton}/\left(\text{ha}\;\text{yr}\right)$, *a* = 0.16 yr^−1^, *Q* = 0.18 (relative water content in a product with 18% dry matter), and *d* = 1.8 yr^−1^ (loss rate of water in soil).

Instead of number of firms we will keep track of acreage, meaning the measure of spread of our hypothetical technology will be the number of hectares grassed.

According to the US Department of Agriculture National Agricultural Statistics Service [76], the average rate to rent cropland in the United States in 2020 was USD 139 per acre (USD 343.5 per ha). The same statistical agency provides us with an information on the annual production of feed grass. In 2019, American farmers produced 82.294 million tons (74.656 million metric tons) of hay and haylage (dry basis), which amounted to USD 19.653 billion. Hence, a selling price for this commodity was USD 263 per ton. So, one ton of feed grass is worth 263/343.5 = 0.77 hectares of rented land. Assuming that only about 40 percent of revenues can be invested in expansion of production, we get *h* = 0.31 ha/ton for the conversion factor of our model.

Equipped with the above numerical values we are now able to estimate the parameters *q*, *γ* and *K* to be 0.59 ton/ha, 6.1 yr^−1^ and 125 ton/ha, respectively.

Using the linked-farm approach of following the same farms in the US agriculture over time, an average annual exit rate of 8.5% is estimated for 2007 to 2012 [77]. This corresponds to a per farm frequency of exits *D* = −ln(1 − 0.085)/(1 yr) = 0.089 yr^−1^.

**Fig 7:**
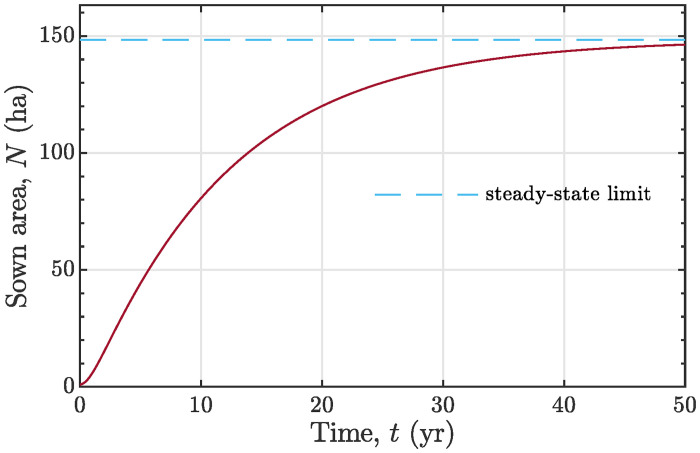
A hypothetical water-limited development of grassed acreage simulated with model (19). The total sown area tends via an *S*-curve to a steady-state size 148.4 ha. The initial conditions are *R*(0) = 0 and *N*(0) = 1 ha; water supply velocity is chosen to be *r* = 500 ton/yr. The other parameters of the model are given in the text.

The numerical integration of Eq (19) with the obtained parameter values yields the diffusion curve shown in Fig 7.

We do not claim that the foregoing numerical example provides an empirical validation of the model. We merely aim at a plausible illustration of our model using a concrete empirical data set.

### Competition in technocenosis

#### The Red Queen hypothesis

Since the time of Darwin, ecologists have believed that changes in the physical and chemical factors of the environment are the biggest single driver of evolution. If the characteristics of the environment were constant, the pace of evolution would almost freeze, but could immediately come alive as external conditions changed.

In the early 1970s Van Valen [78] suggested that an evolutionary acquisition by any species from a given community or an intrusion of a new species into the ecosystem would perceived by all other competitors as an effective change in the environment. To restore broken parity and remain in the niche occupied, the competing species, *nolens volens*, are forced to evolve by fixing their mutations proved to be useful. The ecosystem as a whole gets thrown into an evolutionary progression: some species become extinct while the others change. Thus, evolution can occur even under conditions of unchanging abiotic factors. This idea was termed the *Red Queen hypothesis*, a reference to the world of Lewis Carroll’s Red Queen in “Through the looking-glass and what Alice found there”, who, in order to even stay in the same place, had to run as fast as she could [79, ch. 2]. By now, the Red Queen hypothesis has gained many supporters and has received serious experimental and paleontological evidence. Within the framework of our model for competing technologies (18), it seems interesting to discuss implications of the Red Queen hypothesis as it is applied to evolution of a technocenosis.

Positive steady state in the system (18) requires its right-hand sides to vanish:

$$\begin{array}{c} \sum_{j=1}^{n}q_{ij}N_{j}\;\text{min}(\frac{\gamma_{1j}R_{1}}{R_{1}+K_{1j}N_{\Sigma }},\ldots ,\frac{\gamma_{mj}R_{m}}{R_{m}+K_{mj}N_{\Sigma }})+d_{i}R_{i}=r_{i},\\ \;i=1,\ldots ,m; \end{array}$$ (26a)

$$\begin{array}{c} \text{min}(\frac{\gamma_{1j}R_{1}}{R_{1}+K_{1j}N_{\Sigma }},\ldots ,\frac{\gamma_{mj}R_{m}}{R_{m}+K_{mj}N_{\Sigma }})=D_{j},\\ \;j=1,\ldots ,n;\;N_{\Sigma }=\sum_{j=1}^{n}N_{j}. \end{array}$$ (26b)

Note that populations enter each of the equations (26b) simply as the total number, which can be regarded as a parameter. The subsystem (26b) is independent and contains *n* equations in *m* unknowns. If there is a unique solution we must have

$$\begin{array}{c} \underset{\text{(technologies)}}{n}⩽\underset{\text{(resources)}}{m}, \end{array}$$ (27)

otherwise the subsystem would be overprescribed. In economics terms this inequality means that the number of coexisting technologies in a steady-state technocenosis cannot exceed the number of limiting resources.

Thus, successful introduction of a new technology into the technocenosis while all the incumbents still persisting is impossible without assimilation of a new resource. Indeed, “…it takes all the running you can do, to keep in the same place” [ibid.].

The criterion (27) is only a necessary but not sufficient condition for the existence of an interior fixed point. Additional sufficient conditions depend on the regime of resource supply. Moreover, bounding the number of different resources from below is only needed for coexistence in a stable steady state. Generally, it is possible that the interior fixed point is unstable, and all solutions are attracted to an interior periodic orbit, thus satisfying the definition of so-called *persistence* (e. g., [80]).

#### Competition for the same limiting resource

The general criterion of technology coexistence (27) does not specify which technologies and in what exact number can coexist if a set of resources is fixed. Neither does it predict which technologies will become entrenched in the economic system as a result of competition, if the initial number of competitors exceeds the number of resources.

Consider the competition of *n* technologies for single limiting resource. The corresponding growth equations have the form

$$\begin{array}{c} \begin{array}{cc} \dot{R} & =r-\sum_{j=1}^{n}q_{j}N_{j}G_{j}-dR,\\ {\dot{N}}_{j} & =N_{j}\left(G_{j}-D_{j}\right),\;j=1,\ldots ,n, \end{array} \end{array}$$ (28)

where *G*_*j*_ = *γ*_*j*_ *R*/(*R* + *K*_*j*_ *N*_Σ_) is the growth function and $N_{\Sigma }=\sum_{j=1}^{n}N_{j}$. As before, we will assume that *γ*_*j*_ > *D*_*j*_ for all *j*s.

When discussing an isolated technology at equilibrium, we introduced the break-even resource availability, S*, given by Eq (24). It is convenient to distinguish S* for different technologies *j* by subscripts. And to emphasize that this S* refers to a particular system, namely that containing only technology *j*, we let $S_{\left(j\right)}^{*}$ be the value of S* characterizing technology *j*.

According to the criterion (27), only one technology is expected to survive in the system (28). By comparing break-even availabilities $S_{\left(j\right)}^{*}$ for all *n* competitors it is possible to determine which one of those will win out in the end. Indeed, consider joint dynamics of a pair of technologies, *N*_*k*_ and *N*_*l*_, consuming common resource:

$$\begin{array}{c} \begin{array}{cc} \dot{R} & =r-q_{k}N_{k}G_{k}-q_{l}N_{l}G_{l}-dR,\\ {\dot{N}}_{k} & =N_{k}\left(G_{k}-D_{k}\right),\\ {\dot{N}}_{l} & =N_{l}\left(G_{l}-D_{l}\right), \end{array} \end{array}$$ (29)

where *G*_*j*_ = *γ*_*j*_ *R*/(*R* + *K*_*j*_(*N*_*k*_ + *N*_*l*_)).

If $S_{\left(k\right)}^{*}$ is different from $S_{\left(l\right)}^{*}$ then there is no interior fixed point, that is, a steady state with both *N*_*k*_ and *N*_*l*_ positive. The case of exactly balanced parameters, $S_{\left(k\right)}^{*}=S_{\left(l\right)}^{*}$, is highly unlikely and cannot be expected to be found in reality.

The trivial steady-state (*r*/*d*, 0, 0) is unstable owing to its eigenvalues being λ_1_ = −*d* < 0, λ_2_ = *γ*_1_ − *D*_1_ > 0 and λ_3_ = *γ*_2_ − *D*_2_ > 0.

Besides the trivial equilibrium, two boundary fixed points are possible: $\left({\bar{R}}_{\left(k\right)},{\bar{N}}_{k},0\right)$ and $\left({\bar{R}}_{\left(l\right)},0,{\bar{N}}_{l}\right)$. Let’s find out the necessary and sufficient stability conditions, say, for the first one, at which technology *k* survives and drives technology *l* to extinction. Omitting the details of simple but cumbersome calculations we just mention that it is convenient to express the gain term, *G*_*j*_, in (29) in terms of $S_{\left(j\right)}^{*}$ by making the substitution $K_{j}=S_{\left(j\right)}^{*}\left(\gamma_{j}/D_{j}-1\right)$. Accordingly, the fixed point under consideration will be written as

$$\begin{array}{c} \left(\bar{R},{\bar{N}}_{k},{\bar{N}}_{l}\right)=(\frac{rS_{\left(k\right)}^{*}}{dS_{\left(k\right)}^{*}+D_{k}q_{k}},\frac{r}{dS_{\left(k\right)}^{*}+D_{k}q_{k}},0). \end{array}$$ (30)

Applying the Routh–Hurwitz criterion to fixed point (30) yields its stability condition as $S_{\left(k\right)}^{*}<S_{\left(l\right)}^{*}$. Given this condition, technology *k* approaches a steady-state level, while technology *l* is competitively displaced from the system. This occurs because technology *k* is able to reduce the common resource to such a low level, at which there is insufficient resource for the survival of technology *l*.

**Fig 8:**
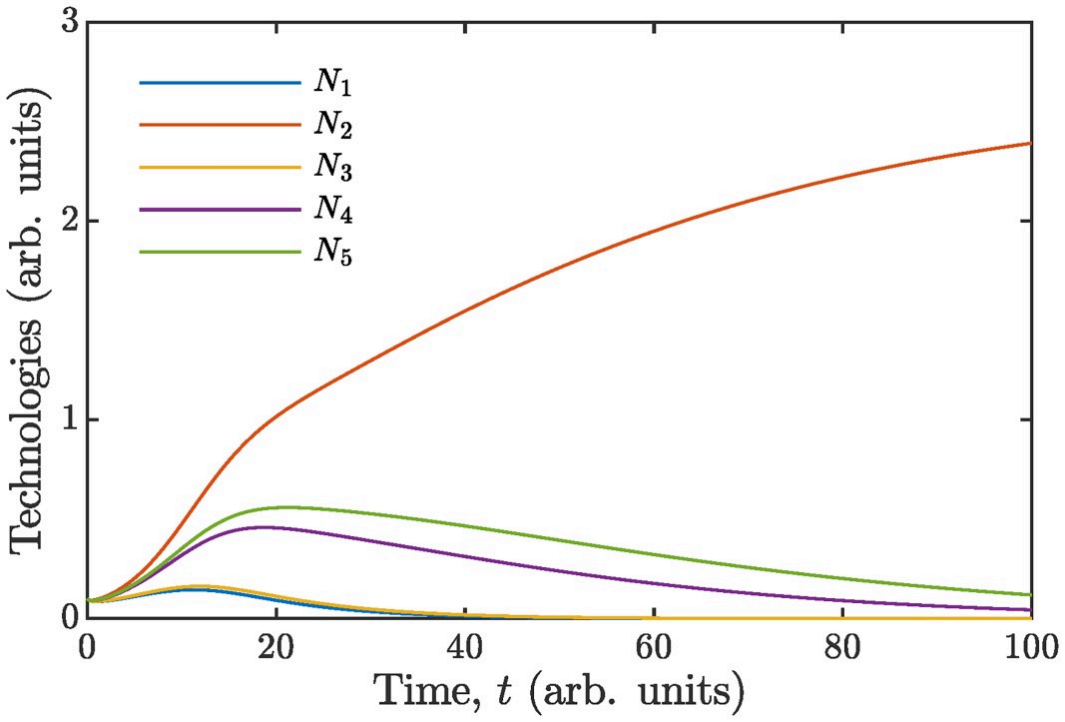
Growth dynamics of five technologies competing for one resource as predicted by Eq (28). Parameters of simulation (arb. units): **K** = (1.02 0.9 1.09 1.17 0.91)^⊤^, *γ* = (0.95 1.1 0.81 0.88 0.98)^⊤^, **q** = (1.04 0.83 0.97 0.84 1.2)^⊤^, **D** = (0.62 0.5 0.61 0.54 0.53)^⊤^, *r* = 2, *d* = 0.2, *R*(0) = 0, and **N**(0) = (0.1 0.1 0.1 0.1 0.1)^⊤^. Because $S_{\left(2\right)}^{*}=0.75$ happens to be the smallest, technology 2 competitively displaces all other rivals by reducing the resource to a level at which they cannot maintain their business.

By comparing different pairs of technologies, we conclude that, when *n* technologies compete for the same limiting resource, the one technology, *s*, with the lowest break-even resource requirement,

$$\begin{array}{c} S_{\left(s\right)}^{*}=\underset{j\in \{1,\ldots ,n\}}{\text{min}}S_{\left(j\right)}^{*}, \end{array}$$ (31)

for the limiting resource, should competitively displace all other technologies in the end. A numerical example of such an outcome of competition is given by Fig 8. It is regrettable, however, that no appropriate real data set is available at the moment compelling us to resort to the help of mockup data.

In the considered problem, the efficiency of a technology *j* can be defined as the reciprocal of its break-even resource availability, $S_{\left(j\right)}^{*}$, given by formula (24). Thus the greater is the ratio of the maximum growth rate to the exit rate (i. e. more favorable is business climate), and the higher is affinity to the resource, the higher will be the efficiency. Statement (31) predicts the direction of technological change in the sense that competition will favor the entrenchment of an innovation whose efficiency value is higher than that of the incumbent competitor.

#### Two technologies and two resources

We shall now proceed to the competition of two technologies for two resources. The starting point is system (18) with *n* = *m* = 2. Because steady states must be within the resource-controlled growth domain in the (*R*_1_, *R*_2_)-plane, two schemes of resource partitioning are possible: (a) both technologies are limited by the same resource, and (b) each competitor is limited by a different resource. The algebraic system of steady-state equations corresponding to ${\dot{N}}_{1,2}=0$ is as follows:

$$\begin{array}{c} \begin{array}{c} \text{min}(\frac{\gamma_{11}S_{1}}{K_{11}+S_{1}},\frac{\gamma_{21}S_{2}}{K_{21}+S_{2}})=D_{1},\\ \text{min}(\frac{\gamma_{12}S_{1}}{K_{12}+S_{1}},\frac{\gamma_{22}S_{2}}{K_{22}+S_{2}})=D_{2}. \end{array} \end{array}$$

Denote a fixed point by $F_{uv}^{xy}$. The upper indices stand for the technologies, the lower ones for the corresponding limiting resources. Absence of a certain upper index at another fixed point means that the technology concerned is extinct.

Suppose both technologies are limited by resource 1. That assumption is equivalent to two simultaneous inequalities to be held in a steady state:

$$\begin{array}{c} \frac{\gamma_{11}{\bar{S}}_{1}}{K_{11}+{\bar{S}}_{1}}<\frac{\gamma_{21}{\bar{S}}_{2}}{K_{21}+{\bar{S}}_{2}},\;\frac{\gamma_{12}{\bar{S}}_{1}}{K_{12}+{\bar{S}}_{1}}<\frac{\gamma_{22}{\bar{S}}_{2}}{K_{22}+{\bar{S}}_{2}}. \end{array}$$

Steady-state coexistence of the technologies under limitation by the same resource 1 would imply

$$\begin{array}{c} \frac{\gamma_{11}{\bar{S}}_{1}}{K_{11}+{\bar{S}}_{1}}=D_{1},\;\frac{\gamma_{12}{\bar{S}}_{1}}{K_{12}+{\bar{S}}_{1}}=D_{2}, \end{array}$$

equivalent to the identity *K*_11_(*γ*_12_/*D*_2_ − 1) = *K*_12_(*γ*_11_/*D*_1_ − 1). This identity would be held over some domain of nonzero measure in the resource plane (*S*_1_, *S*_2_). The occurrence of such a domain is highly unlikely. Hence the interior fixed point $F_{11}^{12}$ corresponding to the scheme of resource partitioning being discussed is not possible. Only boundary steady states $F_{11}^{1\;}$ and $F_{11}^{\;2}$ are allowed.

The necessary condition for $F_{11}^{1\;}$ follows from two simultaneous requirements

$$\begin{array}{c} \frac{\gamma_{11}{\bar{S}}_{1}}{K_{11}+{\bar{S}}_{1}}=D_{1},\;\frac{\gamma_{12}{\bar{S}}_{1}}{K_{12}+{\bar{S}}_{1}}<D_{2}, \end{array}$$

whence we obtain ${\bar{S}}_{1}=S_{1(1)}^{*}<S_{1(2)}^{*}$. Here $S_{i(j)}^{*}$ is the break-even availability of resource *i* for technology *j*, as the reader knows already. In just the same way the respective necessary conditions for three other boundary equilibria, $F_{11}^{\;2}$, $F_{22}^{1}$ and $F_{22}^{\;2}$, where both technologies are limited by the same resource can be found: ${\bar{S}}_{1}=S_{1(2)}^{*}<S_{1(1)}^{*}$, ${\bar{S}}_{2}=S_{2(1)}^{*}<S_{2(2)}^{*}$, and ${\bar{S}}_{2}=S_{2(2)}^{*}<S_{2(1)}^{*}$.

Proceeding to interior steady states, first suppose that technology 1 be limited by resource 1 and technology 2 by resource 2. In a steady state we expect

$$\begin{array}{c} \begin{array}{c} D_{1}=\frac{\gamma_{11}{\bar{S}}_{1}}{K_{11}+{\bar{S}}_{1}}<\frac{\gamma_{21}{\bar{S}}_{2}}{K_{21}+{\bar{S}}_{2}},\\ D_{2}=\frac{\gamma_{22}{\bar{S}}_{2}}{K_{22}+{\bar{S}}_{2}}<\frac{\gamma_{12}{\bar{S}}_{1}}{K_{12}+{\bar{S}}_{1}}. \end{array} \end{array}$$

In terms of break-even resource availabilities these equations lead to

$$\begin{array}{c} {\bar{S}}_{1}=S_{1(1)}^{*}>S_{1(2)}^{*},\;{\bar{S}}_{2}=S_{2(2)}^{*}>S_{2(1)}^{*}. \end{array}$$

The obtained conditions are the necessary requirements for coexistence. They say that one technology must be a superior competitor for one resource and an inferior competitor for the other, and the reverse holds good for the other technology. Limitation of coexisting competitors by different resources imparts more concreteness to the popular verbal phrase “new technology has to find its niche to survive.” Besides, this principle has much in common with the well-known economic *law of comparative advantage* that refers to an agent’s ability to produce goods and services at a lower opportunity cost than that of trade partners.

If technology 1 is limited by resource 2 and technology 2 by resource 1, then the inequality symbols in the coexistence conditions are to be reversed:

$$\begin{array}{c} {\bar{S}}_{1}=S_{1(2)}^{*}>S_{1(1)}^{*},\;{\bar{S}}_{2}=S_{2(1)}^{*}>S_{2(2)}^{*}. \end{array}$$

As will readily be observed, coexistence of two technologies competing for two essential resources imply that the maximum element in either row of the *subsistence matrix* defined as $S=(S_{i(j)}^{*})$ must belong to a different column.

To verify whether one or another predicted interior fixed point represents feasible and stable coexistence, the supply equations should be taken into account. This (lengthy) analysis because of space limitation will not be considered here and will appear elsewhere.

#### Competition of *n* (*n* > 2) technologies for two resources

Now consider *n* populations of firms competing for two nonsubstitutable resources. As we know, at most two competitors may persist in a resulting steady-state technocenosis, because coexisting technologies are to be limited by different resources. The steady-state population equations of the system being considered are

$$\begin{array}{c} \text{min}(\frac{\gamma_{1j}S_{1}}{K_{1j}+S_{1}},\frac{\gamma_{2j}S_{2}}{K_{2j}+S_{2}})=D_{j},\;j=1,\ldots ,n, \end{array}$$

where *γ*_*ij*_ and *K*_*ij*_ are matrices of the dimension 2 × *n*.

The question comes up: what pairs of technologies from the above set of *n* competitors could be potentially eligible for coexistence and under what conditions?

A subsistence matrix associated with the given set of technologies feeding on two resources has the form

$$\begin{array}{c} S=(\begin{array}{cccccccc} S_{1(1)}^{*} & S_{1(2)}^{*} & \cdots & S_{1(k)}^{*} & \cdots & S_{1(l)}^{*} & \cdots & S_{1(n)}^{*}\\ S_{2(1)}^{*} & S_{2(2)}^{*} & \cdots & S_{2(k)}^{*} & \cdots & S_{2(l)}^{*} & \cdots & S_{2(n)}^{*} \end{array}). \end{array}$$ (32)

Let us distinguish two technologies, *k* and *l*, and form their corresponding subsistence matrix:

$$\begin{array}{c} S^{\prime }=(\begin{array}{cc} S_{1(k)}^{*} & S_{1(l)}^{*}\\ S_{2(k)}^{*} & S_{2(l)}^{*} \end{array}), \end{array}$$ (33)

which is, of course, a submatrix of (32). According to the results of our previous analysis, for the pair of *k* and *l* to coexist, the necessary conditions

$$\begin{array}{c} S_{1(k)}^{*}>S_{1(l)}^{*},\;S_{2(k)}^{*}<S_{2(l)}^{*} \end{array}$$

must be held providing technology *k* is limited by resource 1 and technology *l* by resource 2 (otherwise the inequality symbols are to be reversed).

The steady-state equations

$$\begin{array}{c} \frac{\gamma_{1k}{\bar{S}}_{1}}{K_{1k}+{\bar{S}}_{1}}=D_{k},\;\frac{\gamma_{2l}{\bar{S}}_{2}}{K_{1l}+{\bar{S}}_{2}}=D_{l} \end{array}$$

yield the equilibrium levels of the resources:

$$\begin{array}{c} {\bar{S}}_{1}=\frac{K_{1k}}{\gamma_{1k}/D_{k}-1}=S_{1(k)}^{*},\;{\bar{S}}_{2}=\frac{K_{2l}}{\gamma_{2l}/D_{l}-1}=S_{2(l)}^{*}. \end{array}$$ (34)

For the selected pair of technologies, *N*_*k*_ and *N*_*l*_, to coexist against a background of (*n* − 2) intruding competitors, the growth rate of any technology *p* (*p* = 1, …, *n*; *p* ≠ *k*, *l*) at the steady-state resource quantities (34) must be less than the corresponding exit rate:

$$\begin{array}{c} \text{min}(\frac{\gamma_{1p}S_{1(k)}^{*}}{K_{1p}+S_{1(k)}^{*}},\frac{\gamma_{2p}S_{2(l)}^{*}}{K_{2p}+S_{2(l)}^{*}})<D_{p}, \end{array}$$

whence follows

$$\begin{array}{c} \text{max}(S_{1(p)}^{*}/S_{1(k)}^{*},S_{2(p)}^{*}/S_{2(l)}^{*})>1. \end{array}$$

Therefore coexistence of two technologies, *k* and *l*, requires the subsistence square matrix **S**′ (33) of the pair to possess the following properties: (i) maximum element in either row must belong to a different column, and (ii) any column of the augmented subsistence matrix **S** given by (32) (other than columns *k* and *l*) must contain at least one element greater than the maximum entry in the corresponding row of **S**′.

In general, there may be extracted more than one 2 × 2-submatrix with the outlined properties from the augmented subsistence matrix **S**, hence more than one competition outcome could be possible depending on supply of the resources.

#### Competition of *n* technologies for *n* resources (*n* > 2)

Suppose *n* essential resources arrive with constant rates to a given industry of economy, and each of the resources is capable of being a limiting production factor for technologies. Presence of *n* limiting factors in a technocenosis admits coexistence of at most *n* technologies. These technologies must satisfy certain conditions of compatibility. In the foregoing we obtained necessary conditions for the case of *n* = 2, and here follows the general case.

The system of *n* technologies utilizing *n* essential resources is characterized by *n* × *n* subsistence matrix

$$\begin{array}{c} S=(\begin{array}{ccc} S_{1(1)}^{*} & \cdots & S_{1(n)}^{*}\\ \vdots & \ddots & \vdots \\ S_{n(1)}^{*} & \cdots & S_{n(n)}^{*} \end{array}). \end{array}$$ (35)

In a steady state, populations of firms have to obey the conditions of zero growth:

$$\begin{array}{c} \text{min}(\frac{\gamma_{1j}S_{1}}{K_{1j}+S_{1}},\ldots ,\frac{\gamma_{nj}S_{n}}{K_{nj}+S_{n}})=D_{j},\;j=1,\ldots ,n. \end{array}$$ (36)

Assume that all technologies have different coefficients of adaptation, *b*_*ij*_, with respect to each of the resources. This will ensure that minimum cannot fall on the same resource for any pair of equations from system (36). The latter means that no two technologies in a steady state can be limited by the same resource, since otherwise a competitor with higher break-even resource availability will be doomed to extinction.

Assume, for definiteness, that in a steady state, technology 1 is limited by resource 1, technology 2 by resource 2, and so forth. In this case, as imply Eq (36), we get the following set of *n* equations for the equilibrium resource quantities:

$$\begin{array}{c} \frac{\gamma_{jj}{\bar{S}}_{j}}{K_{jj}+{\bar{S}}_{j}}=D_{j},\;j=1,\ldots ,n. \end{array}$$

It can be shown that with such a distribution of limiting factors, each diagonal entry of the subsistence matrix **S** will be maximal in its row. Indeed, let us pick arbitrary nondiagonal elements from, say, the first row of both (*γ*_*ij*_) and (*K*_*ij*_). These will be *γ*_1*k*_ and *K*_1*k*_, respectively, where *k* = 2, …, *n*. Since minimum in the *k*th equation of system (36) falls on resource *k*, an inequality

$$\begin{array}{c} \frac{\gamma_{1k}{\bar{S}}_{1}}{K_{1k}+{\bar{S}}_{1}}>\frac{\gamma_{kk}{\bar{S}}_{k}}{K_{kk}+{\bar{S}}_{k}}=D_{k} \end{array}$$

must be held. Recall, that, on the one hand, ${\bar{S}}_{1}=K_{11}/\left(\gamma_{11}/D_{1}-1\right)=S_{1(1)}^{*}$, and, on the other hand, $K_{1k}/\left(\gamma_{1k}/D_{k}-1\right)=S_{1(k)}^{*}$. For this reason, the above inequality can be rewritten as $S_{1(1)}^{*}>S_{1(k)}^{*}$. Because *k* is chosen arbitrarily, $S_{1(1)}^{*}$ turns out to be the greatest element in the first row of the subsistence matrix (35). Arguing in a similar way with respect to other rows of **S**, we obtain the claimed result.

Thus, coexistence of *n* technologies on *n* limiting resources requires maximum element in each row of the subsistence matrix to come from a different column. Moreover, in a state of long-term coexistence, resource *i* will limit the steady-state growth of a competitor having the greatest break-even threshold for this resource. The noncoincidence of limiting factors mitigates competition to a necessary minimum allowing the technologies to coexist. We discussed the necessary conditions allowing to anticipate the coexistence of competitors. They mean that there must be a certain region in the parametric space of resource supply rates—a *shared niche*—where the interior fixed point exists. The specific location and dimension of the niche are no longer determined by technologies *per se*, but by the supply side. We emphasize that the state of coexistence not necessarily has to be stable. Unstable interior fixed point always implies an accidental choice of one dominant competitor from several alternatives.

#### Competition of *n* technologies for *m* resources (*n* > *m*)

The patterns outlined so far already allow us to anticipate the fate of a technocenosis with an arbitrary number of both technologies and resources. A case of great interest is when the number of contenders to entrench in the technocenosis exceeds the number of resources. An *m* × *n* augmented subsistence matrix

$$\begin{array}{c} S=(\begin{array}{ccccc} S_{1(1)}^{*} & \cdots & S_{1(m)}^{*} & \cdots & S_{1(n)}^{*}\\ \vdots & \ddots & \vdots & \cdots & \vdots \\ S_{m(1)}^{*} & \cdots & S_{m(m)}^{*} & \cdots & S_{m(n)}^{*} \end{array}) \end{array}$$ (37)

can be related to the system of *n* technologies utilizing *m* resources. Since no more than *m* competing technologies can coexist in the system under consideration, the question arises: what combinations from a given set of *n* technologies will coexist and under what specific conditions?

Let us choose, say, the first *m* technologies from *n*. The chosen system will have a square subsistence matrix

$$\begin{array}{c} S^{\prime }=(\begin{array}{ccc} S_{1(1)}^{*} & \cdots & S_{1(m)}^{*}\\ \vdots & \ddots & \vdots \\ S_{m(1)}^{*} & \cdots & S_{m(m)}^{*} \end{array}). \end{array}$$ (38)

As shown earlier, for coexistence of these *m* competitors, the maximum element in each row of matrix (38) must lie in a different column. Suppose, for example, the diagonal elements of matrix **S**′ are maximal in their respective rows, that is, in a steady state, technology *i* is limited by resource *i*.

The steady-state equations

$$\begin{array}{c} \frac{\gamma_{ii}S_{i}^{*}}{K_{ii}+S_{i}^{*}}=D_{i},\;i=1,\ldots ,m) \end{array}$$

yield the equilibrium resource levels

$$\begin{array}{c} {\bar{S}}_{i}=\frac{K_{ii}}{\gamma_{ii}/D_{i}-1}=S_{i(i)}^{*},\;i=1,\ldots ,m. \end{array}$$ (39)

For selected *m* technologies to coexist despite of continuous intrusion activity from the part of (*n* − *m*) strangers, the growth rate of *k*th competitor (*k* = *m* + 1, …, *n*) at resource availabilities (39) must be less than the corresponding exit rate:

$$\begin{array}{c} G_{k}=\text{min}(\frac{\gamma_{1k}S_{1(1)}^{*}}{K_{1k}+S_{1(1)}^{*}},\ldots ,\frac{\gamma_{mk}S_{m(m)}^{*}}{K_{mk}+S_{m(m)}^{*}})<D_{k}. \end{array}$$

This inequality can be represented in the equivalent form

$$\begin{array}{c} \text{max}(S_{1(k)}^{*}/S_{1(1)}^{*},\ldots ,S_{m(k)}^{*}/S_{m(m)}^{*})>1,\;m<k⩽n. \end{array}$$

Therefore, for the chosen first *m* technologies to coexist, their subsistence matrix **S**′ given by (38) is required to have the following properties: (i) maximum element in each row of **S**′ must belong to a different column, and (ii) any column of the augmented subsistence matrix **S** given by (37) (aside from the first *m* columns) must contain at least one entry that happens to exceed the maximum element in a corresponding row of **S**′. The above is true for any subset of *m* technologies of *n*. There may be compiled more than one *m* × *m* submatrix with the outlined properties from the columns of the augmented matrix **S** meaning that several competition outcomes are possible depending on the interplay of resource supply rates *r*_1_, …, *r*_*m*_. In general, for each group of *m* compatible technologies, there are resources whose break-even levels are less than those of any other group, and the resources whose break-even levels are greater than those of any other group. Due to this property, a change in the resource supply may result in displacement of one group of compatible technologies by another, since for each group there is a dedicated shared niche in the space of resource supply rates (*r*_1_, …, *r*_*m*_).

## Discussion

By a number of key features, both economic and biological systems belong to the type of self-organizing:

(i) They are open, continuous-flow systems, exchanging energy, matter and information with the environment. At the microeconomic level of description, the concept of *industrial metabolism* proposed by Ayres [81] seems to be extremely helpful. The word “metabolism” in its original biological meaning characterizes the totality of internal biochemical processes in living organism. An individual cell or the whole organism consumes energy-rich, low-entropy substances to maintain its basic functions, as well as for growth and reproduction. This process is necessarily accompanied by the release of high-entropy waste. Industrial metabolism is an integrated set of physical processes aimed at transforming raw materials, energy, labor and capital into goods and associated waste in a steady mode of operation. The analogy between biological and industrial metabolism is not only about the fact that in both types of systems takes place the conversion of material substances driven by a flow of free energy. For dissipative systems of such a type, relaxation to an equilibrium analogous to “heat death” is impossible: nonzero flows through the system persist even in a steady state;
(ii) Both systems are nonlinear, where autocatalytic processes may occur due to the presence of positive feedback loops. Self-reproduction (self-copying) serves to preserve the information previously created and stored in the system. Economic agents are able to store and hand down managerial and technological information physically recorded in books, databases, technical documentation, software, etc. In biology, at the level of organism, self-reproduction occurs owing to the replication of genetic information recorded in DNA as a sequence of nucleotides. At the same time, biological gene cannot be matched to one unique concept from the economy. An interesting and, in part, controversial question is whether an intangible object can be an example of self-replication. Human cultural traits, such as behaviors, ideas, and technologies that can be learned from other persons undergo transmission and evolution. Models of cultural evolution, both verbal and quantitative, involve concepts from theoretical population genetics and modify them to account for the differences between genetic and cultural transmission [82–84]. Population-based models from ecology are being adapted to study the dynamics of human societies, and optimality modeling can be used to identify behavioral optima as these shift over time and differ among individuals. Dawkins [85] conjectured that elements of self-replication exist in cultural evolution and called these elements *memes*. As examples of memes, he cites melodies, thoughts, slogans, fashionable silhouettes and skills. Imitation and mutual learning play a big role in memes diffusion. The memes hypothesis is rational because it is based on empirical observations. Regardless of whether memes exist, it is now firmly established that self-replication processes play a central role in all higher evolutionary processes;
(iii) Both systems are path-dependent (historically-conditioned), irreversible, in the sense that each economic agent or each organism develops, qualitatively changing in time. Its current state is the result of both dynamic and statistical events. Variability in self-replication is the main source of new information. Hereditary technological information is subject to accidental changes as a result of the heuristic nature of innovation motivated by entrepreneurial activity. The mutational variability of the genetic material is due to irremovable thermal noise affecting enzymatic reactions. Another source of randomness—sexual recombination of genes—has no analog in economic evolution. But in economic development, it is possible to inherit new features acquired through learning, that means Lamarckian evolution. To Boyd et al. [86], “…the cultural evolution of human technology is similar to the genetic evolution of complex adaptive artifacts in other species, like birds’ nests and termite mounds. …Instead the adaptive design evolves gradually in the genetic case through natural selection and in the cultural case by individual learning and biased cultural transmission, with natural selection perhaps playing a secondary role. The big difference between these processes is speed. Cultural evolution is much faster than genetic evolution and, as a consequence, human populations can evolve a variety of tools and other artifacts that are adapted to local conditions. In contrast, most animal artifacts are species-typical adaptations to problems which face all members of the species.”;
(iv) Both systems are hierarchical systems, where each structural level has its own characteristic space-time scale;
(v) Finally, both types of systems share such an important common property of self-organizing systems, as effects of competition. Any emergence of an ordered structure is a result of competition between unstable growing modes: the “surviving” mode suppresses all the rest and imposes its specific structure on the system [87]. The dominance is based on a breakdown of the established parity among the competing agents, caused by the arrival of better adapted mutants. For the dominance to be possible, a limitation should be imposed on the total amount of unorganized or organized matter, or on metabolic inflows and outflows. As a rule, competition occurs for the access to scarce resources (in terms of their stock or flow)—between business entities in economy and between biological populations.

It should be emphasized, however, that it is not the very fact of similarity of economic and biological patterns that is important, but an awareness of the universality of laws of self-organization that lie at their basis.

In the molecular aspect of biological evolution, the emergence of a new species means the emergence of proteins with new functions and new genes corresponding to these proteins. Likewise, in technological change, the emergence of innovation is associated with a new ability to assimilate a different resource or, more formally, with a new form of production function. New technologies explore new sources of resource supply, develop new market niches and adapt to them.

Technological change, interpreted as evolution of technologies through innovation or, in the figurative words of Schumpeter, “the perennial gale of creative destruction”, presupposes economic development in a certain direction. Despite of the well-established fact that invention underlying research and development leading to innovative product or service is a random process [88], the evolution of technologies is not just a random walk in the space of morphological and operating characteristics. The question now is what determines the direction of technological change. As the theory of biological evolution is based on a random variability, which is ultimately governed by mutations and recombination of genomes, so the evolutionary theory of economic change proceeds from innovative entrepreneurial activity expressed in combining factors of production in a new way. No directionality is indicated here. Ordered, progressive and directional evolution is brought about by competition.

In terms of our competition model, dominance means convergence of the system’s trajectory to a particular asymptotically stable boundary fixed point in the space of firm counts. Boundary fixed point is homogeneous (or pure) steady state and is distinguished from highly symmetrical nonhomogeneous equilibria, both trivial and interior, by relatively low degree of symmetry. The appearance of a dominant is always associated with the loss of stability by a homogeneous steady state resulting in a decrease of the symmetry of the system. This is similar to a nonequilibrium second-order phase transition known from statistical physics. The loss of stability is a prerequisite to emergence of new information and thus for establishing of new dominant technology. Broken symmetries are ubiquitous as phenomena and occur in the fields of knowledge as diverse as speciation, morphogenesis, fluid dynamics and stars formation.

Dominance implies the survival of the fittest, best adjusted to the environment. In our model, this type of outcome means converging to a particular stable boundary fixed point. Actually, dominance is predetermined by the fact that the corresponding competitor happens to have the highest efficiency or, equivalently, the lowest break-even resource availability. When there are no interior fixed points, the above condition is always valid, since only one of all present boundary steady states is stable.

The present study has made it possible to obtain the necessary conditions for coexistence of technologies and explain how the technological diversity of an industry depends on the supply of essential resources. The found conditions for coexistence are such that they require the competitors in a steady-state technocenosis to be limited by different resources. In economics, niche is vaguely associated with the existence of market power and barriers to entry (or mobility) of competitors or competitive commodities [89]. The popular thesis “any successful competitor carves out a niche of its own” in the context of our results can be reformulated as follows: each competitor in a steady-state technocenosis has its own limiting factor. The possibility of such a separation of limiting factors is determined not only by differences in the growth functions of the competitors, but also by their exit rates and the relative rates of supply of the resources. We believe that the described mechanism for regulating the diversity of the community of populations of firms is one of the main mechanisms responsible for regulating the technological structure of a real economy.

We already mentioned the interesting similarity between the necessary conditions for coexistence of technologies and the law of comparative advantage. One more analogy should be pointed out here, between the principle of competitive exclusion and the *Tinbergen rule* known since 1952, according to which to successfully achieve *n* independent policy targets at least the same number of independent policy instruments are required. Whether this parallel may turn nontrivial is worth another look.

We wish to remark the role of the resource inflow regime in the formation of the technological structure of the community. The number of technologies in a community cannot exceed the number of resources. Which of the technologies will eventually entrench in the technocenosis depends primarily on the subsistence matrix of all competitors invading the industry and on the relative rates of supply of all essential resources. A change in the rates of resource inflow leads to quantitative restructuring the technological composition of the industry. In a steady-state technocenosis, as the rate of supply of one of the resources increases, the population of a technology that has the greatest break-even threshold for a given resource (i. e. the worst adopted to this resource) also tends to increase, because it is precisely this technology that is limited by the mentioned resource. An excess amount of a certain resource leads to a decrease in the number of operating technologies by one, since the number of limiting factors drops by one. By superimposing the algebraic constrains on break-even resource availabilities derived in this research onto the parametric space of resource supply rates it is possible to get a partitioning of the space into a number of connected regions, closed and open alike, each one representing the specific outcome of competition. A stability analysis of those regions will be presented in the ensuing publication.

Since Hardin [90], much attention in environmental economics has been given to the danger of overexploitation of the common good (e. g., [91]), a situation called the *tragedy of the commons*. A number of mathematical models of this situation has been proposed based on the consumer–resource equations with a properly chosen harvesting strategy function—quota or proportional. One of the most successful minimal models for tragedy of the commons by Bazykin [92, p. 64] accentuates the necessity of assuming the resource to reproduce in a nonlinear fashion. Namely, the behavior of the resource population is assumed to depend drastically on the initial condition: if the initial population density is larger than a certain threshold, the population grows without bound, and it dies out otherwise. According to the model, incorporating the effect of lower critical size of the resource population in the consumer-resource system dooms both populations to extinction for all parameter values. Possible mitigation measures toward the tragedy of the commons include the imposition of private property rights, government regulation, or the endogenous development of a collective action arrangement [93].

On the one hand, the model developed in the present study does not have particular relevance for the tragedy of the commons, as we consider an economic system that is open with respect to resources. The sources of the inputs, as well as the sinks of the unconsumed resources, are not a part of the technocenosis composed of the competing populations of firms, the resources are produced in other industries of the economy, enter the system from outside and do not reproduce themselves within the system. When a single population of firms is considered, it has a nonzero steady-state size whenever the condition *D* < *γ* holds. Otherwise, the population will not be able to survive due to insufficiency of the resource supply. Tragedy of overuse does take place, but in contrast to Hardin’s model, the resource *per se* never becomes depleted irrevocably, no matter what happens to the consumer. On the other hand, in the context of our model, when we consider competition of *n* populations for a particular resource, the condition for their long-term coexistence is the given resource to be limiting factor for only one of the participating competitors. Each of the other (*n* − 1) technologies has to be limited by the own specific resource.

## Conclusion

Mainstream epidemic models of technology diffusion treat this sociologically, as a process by which an innovation is communicated over time among the potential adopters in a social system. Competition between new and incumbent technologies or among multiple contending innovations meant as mutually negative interactions among two or more producers is ignored. The LVG equations formally describe the competition *per se*, but attempts to relate their parameters to mechanisms of resource utilization have not been conclusive. As dissatisfaction with the standard approaches among the economists grows, we, having in mind a desire to develop a more economics-compliant alternative, propose a generic model of technology in the form of a system of consumer–resource Eq (18) with the explicit linkage of resource dynamics, consumption, and growth of population of firms.

Combining the Schumpeter’s vision of production function as unique code for technology with ideas of the evolutionary economics we presume that fund factors of production facilitate the conversion of resources to product in much the same catalytic way as do enzymes in living cells when transforming substrates into different chemical compounds. The production function is shown to be of Leontief–Liebig type in flow-flow representation (17) and plays the role of functional response in the consumer–resource equations mentioned above. However, resources entering the production function are per firm quantities as opposed to substrate concentrations in the Monod trophic function.

Having applied our model to the growth of an isolated technology on a single exhaustible resource (Eq (20)), we succeeded to demonstrate its ability to yield *S*-curves (Fig 2) similar to those generated by conventional models of diffusion (1), (3) and (4). The model is parsimonious yet flexible. The workability of the model was proved by fitting a real dataset for iPod market penetration (Fig 4).

Further, considering a technology growing on the continuous-flow resource we obtained a realistic diffusion *S*-curve (Fig 7) after plugging an empirically justified agricultural production function into Eq (19).

The research agenda proceeds by extending the basic model to include additional resources and competitor populations. A proposition, similar to the ecological principle of competitive exclusion has been proved according to which for a set of competing technologies, which do not interact with one another apart from utilizing the essential resources in common, to coexist in a steady state it is necessary that the number of different resources be not less than the number of competitors.

A population of firms has a steady-state size where growth balances losses, providing its loss rate is less than the maximal rate at which it can grow. The associated steady-state per firm resource quantity is the break-even resource availability S*, which is determined from the technology’s resource-dependent growth function and its loss rate by formula (24). This number is a unique characteristic of technology’s efficiency. When *n* > 1 technologies compete for a single resource this quantity turns to be crucial, and it is shown that in steady state the technology with the lowest $S_{\left(j\right)}^{*}$ excludes all others.

Our study of *n* technologies competing for *n* nonsubstitutable resources revealed that coexistence may be possible providing each technology has its own limiting factor and the maximum element in each row of their subsistence matrix $\left(S_{ij}^{*}\right)$ belongs to a different column.

The number *n* of technologies that might fix in a technocenosis is normally greater than the number *m* of limiting resources. We found what groups of technologies from the set of *n* competitors would coexist and under what terms. The condition requires that any column of the *m* × (*n* − *m*) subsistence matrix for the community of (*n* − *m*) excluded technologies must have at least one element that is greater than the maximal entry in the corresponding row of the *m* × *m* subsistence matrix for the community of *m* technologies winning the competition.

All in all, we believe that the proposed model of competition, which explicitly takes into account the competition of technologies for essential resources, enables to obtain more meaningful economic results in comparison with the existing population-based models of technology diffusion.
